# *In silico* analyses of protein glycosylating genes in the helminth *Fasciola hepatica* (liver fluke) predict protein-linked glycan simplicity and reveal temporally-dynamic expression profiles

**DOI:** 10.1038/s41598-018-29673-3

**Published:** 2018-08-03

**Authors:** Paul McVeigh, Krystyna Cwiklinski, Andres Garcia-Campos, Grace Mulcahy, Sandra M. O’Neill, Aaron G. Maule, John P. Dalton

**Affiliations:** 1 Parasitology & Pathogen Biology, The Institute for Global Food Security, School of Biological Sciences, Queen’s University Belfast, Belfast, UK; 2 School of Veterinary Medicine, University College Dublin, Dublin, Ireland; 3 Department of Biotechnology, Dublin City University, Dublin, Ireland

## Abstract

Glycoproteins secreted by helminth parasites are immunogenic and represent appealing components of vaccine preparations. Our poor knowledge of the pathways that mediate protein glycosylation in parasitic flatworms hinders our understanding of how proteins are synthesised and modified, and our ability to target these pathways for parasite control. Here we provide the first detailed description of genes associated with protein glycosylation in a parasitic flatworm, focusing on the genome of the liver fluke (*Fasciola hepatica*), which is a globally important trematode parasite of humans and their livestock. Using 190 human sequences as search queries against currently available *F*. *hepatica* genomes, we identified 149 orthologues with putative roles in sugar uptake or nucleotide sugar synthesis, and an array of glycosyltransferase and glycosidase activities required for protein N- and O-glycosylation. We found appreciable duplication within these orthologues, describing just 87 non-redundant genes when paralogues were excluded. *F*. *hepatica* lacks many of the enzymes required to produce complex N- and O-linked glycans, which explains the genomic basis for the structurally simple glycans described by *F*. *hepatica* glycomic datasets, and predicts pervasive structural simplicity in the wider glycome. These data provide a foundation for functional genomic interrogation of these pathways with the view towards novel parasite intervention strategies.

## Introduction

*Fasciola* spp. liver fluke are trematode parasites of humans and animals that have global impacts on agriculture, animal welfare, food security and human health^1–4^. These impacts are predicted to increase with climate change and increasingly intensive farming^5^. Wild fluke populations have evolved resistance to four of the five currently available flukicides^6^, highlighting the need for new chemotherapeutics and/or vaccines. Despite sustained research efforts, we still lack a commercially-viable vaccine for the prevention of liver fluke infections^7^, and no new chemical interventions are close to market. New tools and resources are now available for liver fluke that will assist such drug/vaccine control efforts, including a genome, several transcriptomes and various functional genomics toolsets that we, and others, have developed to help streamline liver fluke drug/vaccine discovery pipelines^8–10^.

One explanation for the poor vaccine efficacy of liver fluke antigens in large animal models is our inability to replicate native post-translational modifications, such as glycosylation, using standard recombinant protein expression systems^7^. This technical issue has been compounded by the absence of detailed data on the structure of the glycans that are presented by the parasite within the definitive, mammalian host. Immunochemical studies showed O-linked Tn antigen structures on the surface (tegument) of adult liver fluke^11,12^. Our recent glycomic studies showed that the adult tegument and some secreted proteases bear high-mannose and oligomannose structures, supplemented by a small number of truncated complex and hybrid glycans^13,14^. Although informative, these data may represent only a small proportion of the entire liver fluke glycome, when other tissue types and life stages are considered. In the absence of further targeted proteomic/glycomic datasets, our hypothesis was that the genomic complement of glycosylating enzymes, and their expression patterns across life stages, could be informative of life-stage and tissue variation within the wider glycome. Additionally, the enzymes and transporters within these pathways could represent chemotherapeutic targets through which glycosylation could be interrupted, undermining fluke virulence and survival. This hypothesis is strengthened by the existence of chemical inhibitors of specific proteins within eukaryotic N-glycosylating pathways, and evidence of the importance of surface glycans for efficient penetration of host tissue by *F*. *hepatica*^15^. No data are available on the glycosylating pathways of liver fluke, nor for any flatworm apart from a single study focusing on the fucosylation subset of transferases in *Schistosoma mansoni*^16^. Therefore, this work represents the most in depth study of glycosylation pathways in phylum Platyhelminthes, and the first such study in *F*. *hepatica*.

Our aims for this study were threefold: (i) Exploit liver fluke genome data to advance understanding of the biosynthesis and processing of the largely mannosidic N-glycans that have been reported by mass spectrometry (MS) studies; (ii) Use presence of glycosidase and glycoysyltransferase orthologues to predict the occurrence of more complex N- and O-glycans than those reported by MS studies; and (iii) By analysing changes in expression of glycogene orthologues across fluke developmental RNA-Seq and proteomic datasets, to infer whether protein glycosylation patterns might change during intra-mammalian development. Our data showed that the *F*. *hepatica* glycogenome is characterised by a relative paucity of single orthologues of human glycosylation genes, but that many of those orthologues existed as multiple paralogues, indicating gene duplication. The *F*. *hepatica* glycosylation gene complement adequately explained the largely mannosidic nature of N-glycans reported in MS datasets. The absence of genes coding for enzymes responsible for more structurally complex glycans is notable, suggesting pervasive structural simplicity amongst the N- and O-linked glycoproteins of *F*. *hepatica*. Differential expression of glycogenes across intra-mammalian RNA-Seq and proteomic datasets was apparent, suggesting that glycosylation profiles probably change during fluke development.

## Methods

### Datasets, search methodology and analyses

Query sequences were retrieved from human datasets by a combination of literature searches and via relevant pathways annotated in the UniProt database (www.uniprot.org). All query sequences (Supplementary Dataset S1) were searched by BLASTp against two *F*. *hepatica* genomes hosted by WormBase ParaSite^17^. These were generated by Liverpool University / Queen’s University Belfast (http://parasite.wormbase.org/Fasciola_hepatica_prjeb6687/Info/Index/ ^8^, and Washington University, St Louis (http://parasite.wormbase.org/Fasciola_hepatica_prjna179522/Info/Index/ ^18^. All hits scoring E < 1e-3 (Pearson *et al*., 2013^19^) were retained and confirmed by reciprocal BLASTp against the relevant dataset. Sequences were then retained only where their best reciprocal hit matched one of the original search queries. Where a query sequence lacked a best reciprocal hit, we considered that sequence as lacking an *F*. *hepatica* orthologue. Duplicates were removed based on sequence similarity: multiple *F*. *hepatica* sequences were aligned in Mega v7 (www.megasoftware.net), and trimmed to a core sequence block shared by all members of the alignment. This alignment was converted into a percent identity matrix (with Clustal Omega: https://www.ebi.ac.uk/Tools/msa/clustalo/). Sequences sharing ≥90% identity within this matrix were considered identical, with only the sequence showing the highest BLAST bit score retained as the definitive sequence. Sequences sharing <90% identity but sharing the same best reciprocal BLAST hit were aligned and manually examined for duplication. Where two sequences matched exactly over the whole length of the shortest sequence, only the single sequence with the highest bit score was retained. All remaining sequences were searched for protein domains using either InterProScan https://www.ebi.ac.uk/interpro/search/sequence-search) or HMMSCAN (https://www.ebi.ac.uk/Tools/hmmer/search/hmmscan). Relevant information on the function and catalytic activity of each orthologue (as described in the UniProt database) was also retained (Supplementary Dataset S2).

Where orthologues were absent from *F*. *hepatica* (e.g. sialic acid related genes), we performed additional hidden Markov model (HMM)-driven searches, using hmmer 3.0 (http://hmmer.org/). Within hmmer, the *hmmbuild* module was used to create an HMM from a protein multiple sequence alignment, which was subsequently searched against *F*. *hepatica* proteins using the *hmmsearch* module. For these genes, we also performed tBLASTn searches against *F*. *hepatica* genome scaffolds to confirm their absence. In all cases, these HMM and tBLASTn searches agreed with our original BLASTp calls, supporting the absence of orthologues.

Protein multiple sequence alignments (MSA) were generated in MAFFT (https://mafft.cbrc.jp/alignment/server/). Alignments were trimmed in Mega 7 (http://www.megasoftware.net/) to produce contiguous sequence blocks before being used to generate maximum likelihood (ML) phylogenetic trees via PhyML (http://www.phylogeny.fr/). Statistical support for branches was obtained using the approximate likelihood-ratio test ^20^, using default (SH-like) parameters.

Circos diagrams were generated using Circos Table Viewer online (http://mkweb.bcgsc.ca/tableviewer/visualize/). Each Circos diagram was generated from a data table containing the exponential value of the E-value comparison from BLASTp searches between *F*. *hepatica* sequences and the human genome (NCBI BLASTp vs UniProt dataset, restricted to *Homo sapiens*). All tools used default parameters.

### RNAseq and proteomic analyses

As part of our analysis we exploited previously published datasets describing: (i) Developmentally-staged RNAseq datasets representing *F*. *hepatica* metacercariae, newly excysted juveniles (NEJs) maintained *in vitro* for 1 h, 3 h or 24 h post excystment, *ex vivo* parasites recovered from rat liver parenchyma 21-days post infection, and adult parasites recovered from cattle bile ducts at abattoir^8,9^; (ii) Proteomic datasets representing N-glycoproteins from the adult tegument, excretory-secretory (ES) products from adults and NEJs, extracellular vesicles (EVs) from NEJs and somatic extracts from metacercariae and NEJs^8,9,14^. These datasets were processed and analysed as previously described^8,9,14^.

### Data availability

All data generated or analysed during this study are included in this published article (and its Supplementary Dataset files).

## Results and Discussion

**Figure 1:**
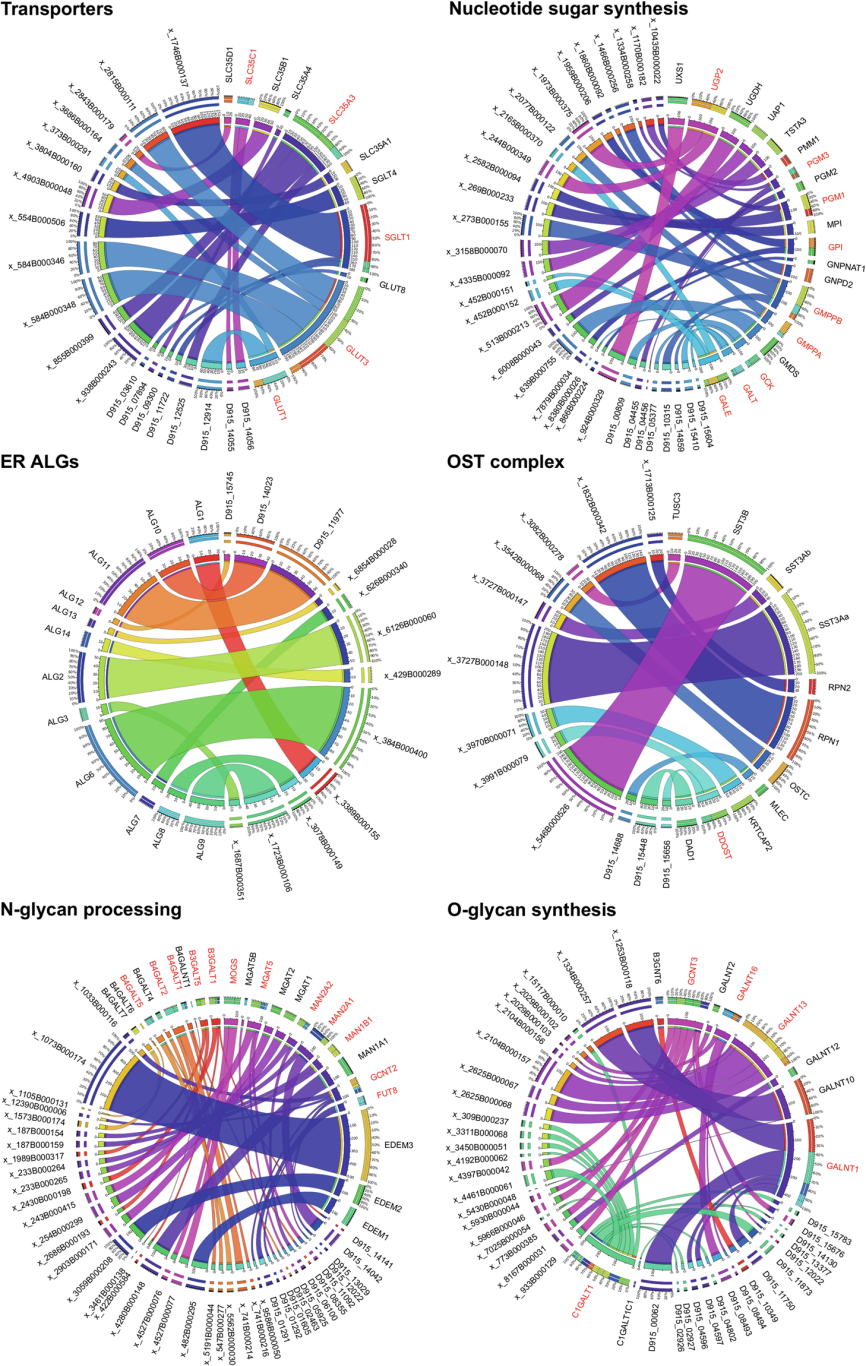
Gene duplication and multiple paralogues of *Fasciola hepatica* glycosylation genes. Circos diagrams illustrating best reciprocal hit orthologues of human glycosylating genes in *F*. *hepatica*. Genes are presented as functional groups: (i) Transporters (including glucose and nucleotide sugar transporters); (ii) Nucleotide sugar synthesis; (iii) Endoplasmic reticulum altered glycosylation mutant genes (ER ALGs); (iv) Oligosaccharyltransferase (OST) complex; (v) N-glycan processing; (vi) O-glycan synthesis. Gene names are indicated around the circumferences, including human and *F*. *hepatica* (latter labels beginning D915_, or x_, where “x_” substitutes for “BN1106_s” in reference to the *F*. *hepatica* genomes PRJNA179522 and PRJEB6687, respectively^17^). The width of ribbons linking genes is determined by the exponent of the BLAST e-value score (Supplementary Dataset S2) where a wider ribbon indicates a higher scoring match. Gene names in red text indicate genes with multiple *F*. *hepatica* paralogues.

We identified a total of 149 *F*. *hepatica* genes relating to protein glycosylation pathways, as detailed in Supplementary Datasets S2 and S3. These represent orthologues of 190 human query sequences, and below we define each *F*. *hepatica* gene in terms of its closest human orthologue. Table 1 describes the human and liver fluke datasets in terms of functional category. The *F*. *hepatica* gene total incorporated many examples of gene duplication, where multiple *F*. *hepatica* paralogues shared a single human gene as their best reciprocal BLAST hit. When these paralogous gene sets were collapsed to form a “non-redundant” dataset, the *F*. *hepatica* complement fell to just 87 genes, illustrating reduced functional complexity in liver fluke glycosylating pathways relative to that seen in human. Figure 1 shows that paralogues identified included: 13 for C1GALT1; six for GCNT3; five for each of GALNT1 and GALNT13; four for each of GCNT2 and GLUT3; three for B3GALT1, GALE, GALNT16, GALT, GMPPB, MGAT5, SGLT1 and SLC35A3; and, two for each of B3GALT5, B4GALT1, B4GALT2, B4GALT5, DDOST, FUT8, GCK, GLUT1, GMPPA, GPI, MAN2A2, MAN1B1, MOGS, PGM1, PGM3, SLC35C1 and UGP2 (Fig. 1; for definitions of these genes, see Supplementary Dataset S2).

**Table 1:** Glycosylating gene categories and numbers of genes in human vs *Fasciola hepatica*.

Functional category	*Homo sapiens* genes	*Fasciola hepatica* total genes (including paralogues)	*Fasciola hepatica* non-redundant orthologues
Monosaccharide transport	18	11	5
Nucleotide sugar synthesis	24	32	19
Nucleotide sugar transport	6	9	6
ER ALGs	12	12	12
OST complex	12	12	11
N-glycan processing	91	37	23
O-glycan processing	27	36	10
Total	190	149	87

Gene duplication in the *F*. *hepatica* genome is now a familiar concept^8^, but mining the comparative genomics resources available on WormBase ParaSite^17^ suggests that most of the multiple paralogous genes we report here also show multiplicity in other flatworms (Supplementary Dataset S4). To take just one example, multiple orthologues of Glycoprotein-N-acetylgalactosamine 3-beta-galactosyltransferase 1 (C1GALT1; responsible for synthesis of Core 1 O-glycan structures) appear in 28 flatworm genomes, at an average of five genes per species. C1GALT1 multiplicity is also seen in nematodes, where orthologues are apparent in 52 species at an average of four genes per species. Ten non-helminth genomes are represented amongst the WormBase ParaSite comparative genomics datasets; in most of these genomes, C1GALT1 orthologues occur as a single gene per species. Based on these datasets, C1GALT1 gene duplication appears to be a helminth-based phenomenon, albeit the functional consequences of this multiplicity are unclear. In flatworms generally, we do not yet have functional data for any of these paralogues, but since individual *F*. *hepatica* C1GALT1 paralogues generally share less than 70% sequence identity, we can speculate that the trajectory of this divergence is towards functional separation. While we cannot predict what impact, if any, this would have on glycosylation phenotype, it opens an interesting avenue for further work to address this question. Although individual gene paralogues appear on distinct genome scaffolds, the currently fragmented status of available *F*. *hepatica* genomes makes it impossible to discern the relative chromosomal positions of each paralogue within the genome^8,18^.

### Monosaccharide transporters

**Figure 2:**
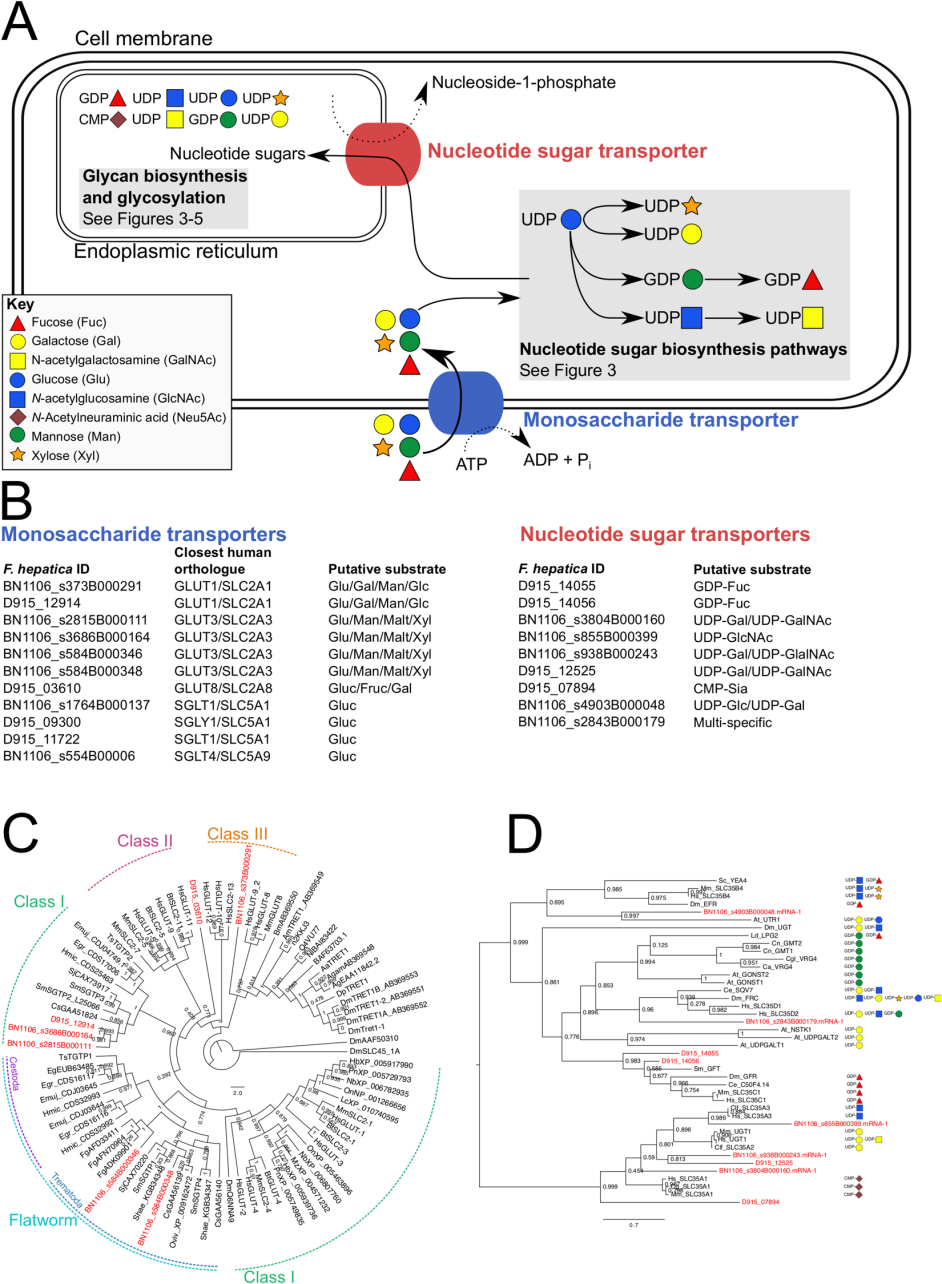
Transmembrane transport of monosaccharides and nucleotide sugars. (**A**) Schematic of predicted sugar transport capacity based on homology of *Fasciola hepatica* transporters with characterised human monosaccharide transporters (responsible for cellular transport of sugars across the plasma membrane) and nucleotide sugar transporters (which carry nucleotide sugars across the Golgi membrane). (**B**) The *F*. *hepatica* genome contains coding sequences for eleven monosaccharide transporters, and nine nucleotide sugar transporters. The closest human orthologues and their substrate transport capacities are indicated. (**C**) Maximum Likelihood phylogeny of *F*. *hepatica* glucose transporters (GLUT), alongside orthologues from flatworms, invertebrates and vertebrates. *F*. *hepatica* sequences are highlighted in red text. Circumference labels denote three established phylogenetic divisions of GLUTs^20^, as well as the clade of flatworm GLUTs described by Cabezas-Cruz *et al*.^39^. (**D**) Maximum Likelihood phylogeny of *F*. *hepatica* nucleotide sugar transporters, alongside orthologues from flatworms (*Schistosoma mansoni* Sm_GFT) and other organisms, modified from^15^. *F*. *hepatica* sequences are highlighted in red text. The substrate of each transporter is shown where established. Node labels (**C**,**D**) indicate statistical support yielded by approximate likelihood ratio test (aLRT). 2C, 2D Species abbreviations: Aa, *Aedes aegypti*; Agam, *Aedes gambiae*; Am, *Apis mellifera*; Bm, *Bombyx mori*; Bt, *Bos taurus*; Cs, *Clonorchis sinensis*; Dp, *Drosophila pseudoobscura*; Dm, *Drosophila melanogaster*; Egr, *Echinococcus granulosus*; Emuj, *Echinococcus multilocularis*; Fg, *Fasciola gigantica*; Hb, *Haplochromis burtoni*; Hmic, *Hymenolepis microstoma*; Hs, *Homo sapiens;* Lc, *Larimichthys crocea*; Mm, *Mus musculus*; Mz, *Maylandia zebra*; Nb, *Neolamprologus brichardi*; On, *Oreochromis niloticus*; Oviv, *Opisthorchis viverrini*; Pn, *Pundamilia nyererei*; Shae, *Schistosoma haematobium*; Sj, *Schistosoma japonicum*; Sm, *Schistosoma mansoni*; Ts, *Taenia solium*.

Glucose is the primary source of the cellular monosaccharides that are used for biosynthesis of nucleotide-linked sugars^21^. Nucleotide sugars are subsequently imported to the ER by specific transporters, and combined into polysaccharide chains during glycan synthesis, (both of these processes are described in the relevant sections, below). Cellular absorption of glucose from the extracellular environment is mediated by glucose transporters; in humans, these transporters are encoded by genes GLUT1–14, which are members of the SLC2 gene family^22^. Each GLUT is a membrane protein bearing 12 transmembrane domains. The *F*. *hepatica* genome contained seven GLUT-like sequences (Fig. 2; Supplementary Dataset S2), all of which contained Pfam domain PF00083 (sugar (and other) transporter), and at least six transmembrane domains. Human GLUTs form three phylogenetic clades designated Class 1 (GLUT1-4, GLUT14), Class 2 (GLUT5, GLUT7, GLUT9, GLUT11) and Class 3 (GLUT6, GLUT8, GLUT10, GLUT12)^22^. The majority of GLUTs are well-characterised in humans, permitting us to map our putative orthologues in *F*. *hepatica* against these established classes. The *F*. *hepatica* GLUT complement (Fig. 2A–C) included: (i) two orthologues of GLUT1, a transporter of glucose, as well as galactose, mannose and glucosamine that is ubiquitously expressed in humans^22,23^; (ii) four orthologues of GLUT3, a facilitative transporter of glucose, as well as mannose, maltose, xylose and dehydroascorbic acid that is expressed predominantly in the human brain, as well as in placenta, spermatozoa, and pre-implantation embryos;^22,24–26^ and (iii) one orthologue of GLUT8, a predominantly testis-expressed transporter^27^ that appears to be retained intracellularly^28,29^, but transports glucose, fructose and galactose when heterologously expressed at the surface of *Xenopus* oocytes^27^. In addition to GLUT-like facilitated transporters, we also identified orthologues of the sodium-dependent glucose transporters SGLT1/SLC5A1 and SGLT4/SLC5A9 (Supplementary Dataset S2). Based on the known activities of the human orthologues, we propose that *F*. *hepatica* can absorb a range of hexose monosaccharides, although key functional studies, such as (i) quantification of the sugar transport capacity of *F*. *hepatica* GLUTs within a heterologous expression system and (ii) analysing the phenotypic consequences of silencing *F*. *hepatica* GLUTs on worm growth, development and survival, will be required to confirm this hypothesis.

In flatworms, sugar transporters have been described in trematodes (*Clonorchis sinensis*, *S*. *mansoni*) and cestodes (*Taenia* spp.)^30–40^, with the four *S*. *mansoni* transporters (SGTP1-4) being the most intensively studied^30,32,37^. Two of these (SGTP1 & SGTP4) have been studied using RNA interference (RNAi), where knockdown of either one or both genes impaired glucose uptake, leading to reduced survival of parasites maintained in glucose-depleted media^37^. A separate study showed differential regulation of SGTP transcripts in the presence of glucose: SGTP1 & 4 were downregulated by 10 mM glucose, while SGTP2 & 3 were upregulated^39^. The latter authors further reinforced these pairings by showing that: (i) phylogenetically, SGTP2 & 3 group together with vertebrate Class 1 transporters, while SGTP1 & 4 reside within a flatworm-only clade of transporters; and (ii) SGTP2 & 3 and SGTP1 & 4 show distinct affinities for glucose. In the former group, SGTP2 lacks glucose transport capacity^30^. Our phylogeny (Fig. 2C) reflects that of Cabezas-Cruz *et al*.^39^, placing three *F*. *hepatica* GTPs within Class I (D915_12914, BN1106_s3686B000164, BN1106_s281B000111) alongside *S*. *mansoni* SGTP2 and SGTP3. Two *F*. *hepatica* transporters resolved within a flatworm-only clade (BN1106_s584B000346, BN1106_s584B000348) alongside *S*. *mansoni* SGTP1 and SGTP4, and the *C*. *sinensis* glucose transporter CsGLUT^40^. Further transporters appeared within our phylogeny, one in in Class III (BN1106_s373B000291), and one residing between Classes II and III (D915_03610). These data suggest that the glucose transport capacity of *F*. *hepatica* likely overlaps that of *S*. *mansoni.* However, the biological consequence of the expanded complement of sugar transporters in *F*. *hepatica*, relative to *S*. *mansoni*, remains to be elucidated.

### Synthesis of nucleotide sugars

**Figure 3:**
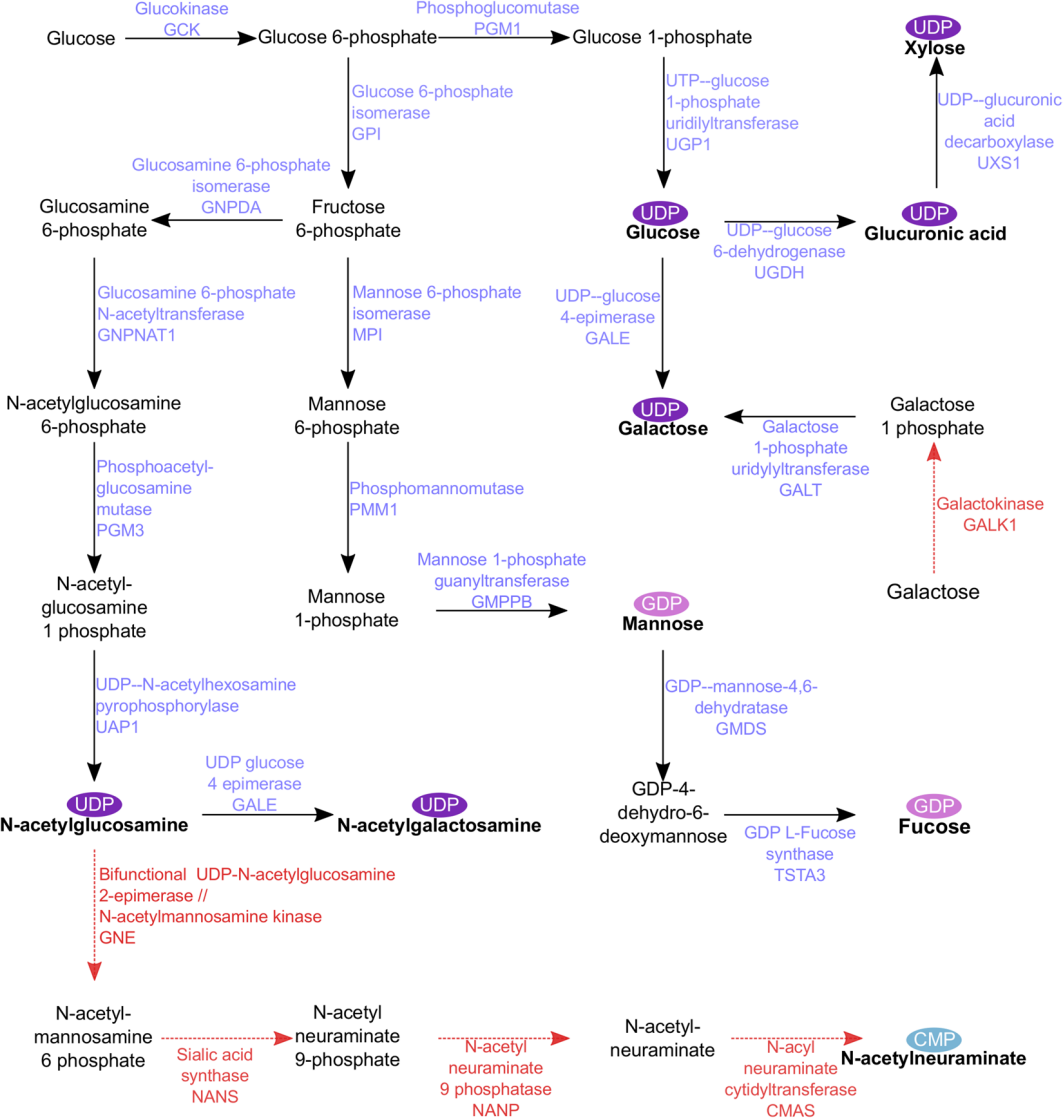
Nucleotide sugar biosynthesis. Precursors, intermediates and enzymes involved in the biosynthesis of nucleotide sugars. Nucleotide sugars are shown in bold, appended with CMP, GDP or UDP. Enzymes are shown in purple where we identified an orthologue in the *Fasciola hepatica* genome, and red where we did not. Each named enzyme is followed by the associated human gene name. For full annotation data, see Supplementary Dataset S2.

Glycans are synthesised from high-energy activated monosaccharide donors called nucleotide sugars, which are formed by a reaction between a monosaccharide and a nucleoside triphosphate. In the context of glycobiology these include CMP-*N*-acetylneuraminic acid/sialic acid (CMP-Neu), GDP-Fucose (GDP-Fuc), GDP-Mannose (GDP-Man), UDP-Glucose (UDP-Glc), UDP-Galactose (UDP-Gal), UDP-Glucuronic acid (UDP-Gla), UDP-*N*-acetylgalactosamine (UDP-GalNAc) UDP-*N*-acetylglucosamine (UDP-GlcNAc) and UDP-Xylose (UDP-Xyl), all of which can be produced from a glucose or other hexose sugar precursor, within a single biosynthetic pathway (Fig. 3). This pathway enables formation of each nucleotide sugar by *de novo* synthesis (reaction between a glycosyl-1-phosphate and a nucleoside triphosphate), while some can also be formed by conversion from another activated monosaccharide. No data are available on these biosynthetic genes in flatworms aside from the fucosylation-associated genes reported in *S*. *mansoni*^16^. This study therefore represents the first comprehensive description of nucleotide sugar biosynthesis genes in any flatworm parasite. The mammalian pathway contains 24 enzymes, 19 of which have identifiable orthologues in the *F*. *hepatica* genome (Fig. 3; Supplementary Dataset S2). We were unable to identify orthologues of galactokinase (GALK1), nor of any of the four sequentially-acting enzymes associated with sialic acid (N-acetylneuraminic acid) synthesis from *N*-acetylglucosamine (GNE, NANS, NANP, CMAS; Fig. 3). This suggests that, with the exception of sialic acid, *F*. *hepatica* has the machinery to synthesise nucleotide sugars *de novo*, rather than having to obtain them from the host (although they could potentially absorb nucleotide sugars from the contents of lysed host cells). The absence of the sialic acid biosynthesis pathway is consistent with the absence of sialylated glycoproteins in glycomic studies of *F*. *hepatica*^13,14^; although sialic acid was detected in the latter study its presence was attributed to contamination by host glycans. Sialylation also appears absent from schistosome glycoproteins^41–44^, although binding of sialic acid lectins has been described in the cestodes *Echinococcus granulosus* and *Taenia solium*^45,46^. The general view across invertebrates is that sialic acids are only seen in glycans of the deuterostome lineage^47^. We hypothesise that *F*. *hepatica*, like other protostome invertebrates, does not glycosylate with sialic acid.

### Nucleotide sugar transport

Nucleotide sugar biosynthesis occurs in the cytosol, while glycan synthesis occurs in the ER and Golgi, as described below. This physical separation requires the transport of nucleotide sugars across the Golgi membrane into the lumen before they can be used in glycan synthesis. Trans-membrane transport is mediated by energy-independent nucleotide sugar transporters (NSTs) that, as antiporters, couple the inward transport of nucleotide sugars into the ER lumen to outward flow of nucleoside monophosphates. Prior to this study, there was no information concerning NSTs in flatworms beyond a single study describing one GDP-Fucose transporter in *S*. *mansoni* (SmGFT^16^). We have identified nine NSTs in the *F*. *hepatica* genome (Fig. 2B). Peterson *et al*. employed phylogenetic comparisons with a broad range of other NSTs to classify SmGFT, in line with previous work showing that NSTs can be accurately separated into functional groups by phylogenetic analyses^48,49^. Many of the clades revealed by these studies included closely-related NSTs with aberrant substrate specificities^16^. We have adapted Peterson’s analysis to incorporate the nine NSTs identified from the *F*. *hepatica* genome (Fig. 2A,D). This analysis suggested that *F*. *hepatica* possesses transporters similar to those with specificity for UDP-Gal and UDP-Glc (BN1106_4903B000048), GDP-Fuc (D915_14055, D915_14056; these are the closest orthologues to SmGFT), UDP-GlcNAc (BN1106_s855B000399), UDP-Gal and UDP-GalNAc (BN1106_s938B000243, BN1106_s3804B000160, D915_12525) as well as CMP-Neu (D915_07894; significant given the absence of other sialic acid-related components from the rest of these pathways). The remaining *F*. *hepatica* NST most closely resembled a clade of multi-specific transporters carrying UDP-Gal, UDP-GalNAc, UDP-Glc, UDP-GlcNAc, UDP-Xyl and GDP-Man. Several classes of SLC transporters are targeted by drugs in human medicine^50^ and further research could designate helminth NSTs as valid targets for future anthelmintic screening strategies.

### N-glycan synthesis

**Figure 4:**
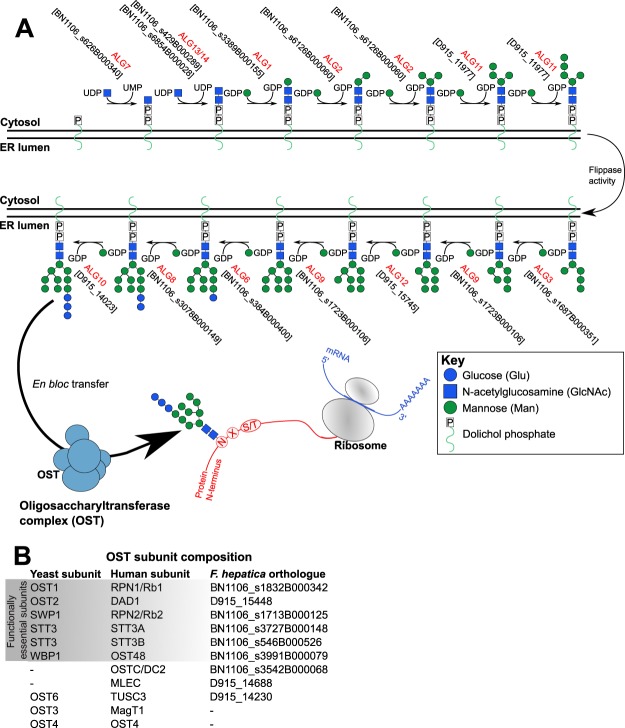
N-glycan biosynthesis within the Endoplasmic Reticulum (ER). **(A**) Initial stages of N-glycan synthesis involve the production of a 14 sugar (Man_9_Glc_3_GlcNAc_2_) precursor glycan, which is constructed onto a dolichol phosphate anchored in the ER membrane. This process is catalysed by the sequential actions of twelve ALG (named for their altered glycosylation phenotype in knockout yeast) glycosyltransferases, each of which has an orthologue in the *F*. *hepatica* genome (accessions indicated in square brackets). Initial steps occur on the cytosolic surface of the ER membrane, with latter stages occurring on the luminal surface. (**B**) Subunit composition of the oligosaccharyltransferase (OST) complex in human, yeast and *F*. *hepatica* orthologues of each.

N-linked glycoprotein synthesis is coupled to a highly conserved series of enzymatic reactions occurring on the ER membrane. These produce a 14-sugar precursor glycan (Man_9_Glc_3_GlcNAc_2_) linked to a membrane-anchored dolichol phosphate. This precursor glycan is subsequently transferred *en-bloc* onto a newly synthesised protein. Our understanding of this process in eukaryotes is based largely on experiments performed in mutant yeast, where the Man_9_Glc_3_GlcNAc_2_ glycan is constructed by the sequential actions of twelve ALG (altered glycosylation mutant) genes^21^. This process is conserved across Eukaryota^21^ and orthologues of all twelve ALGs can be identified in the *F*. *hepatica* genome (Fig. 4A). These data confirm that *F*. *hepatica* is likely capable of generating the Man_9_Glc_3_GlcNAc_2_ precursor glycan on the ER membrane, through a mechanism that is probably identical to that seen in other metazoans.

Transfer of the precursor glycan from the ER membrane onto a newly-synthesised protein is performed by a multimeric enzyme complex called oligosaccharyltransferase (OST). OST transfers Man_9_Glc_3_GlcNAc_2_ from its dolichol phosphate anchor, to an Asn-X-Ser/Thr (X = any amino acid) N-glycosylation motif on a newly-synthesised protein within the ER lumen. Yeast OST contains eight individual protein subunits and human OST contains at least eleven^51,52^. *F*. *hepatica* has orthologues of nine OST subunits (Fig. 4B), including all five that are crucial for cell survival in yeast (OST1, OST2, STT3, SWP1, WBP1). The two subunits that are absent from *F*. *hepatica* are not functionally essential, but are important for OST complex stability and glycosylation activity^51–54^. Collectively, our findings suggest that the subunit composition and function of OST is well conserved in *F*. *hepatica* relative to other eukaryotes.

### N-glycan processing

**Figure 5:**
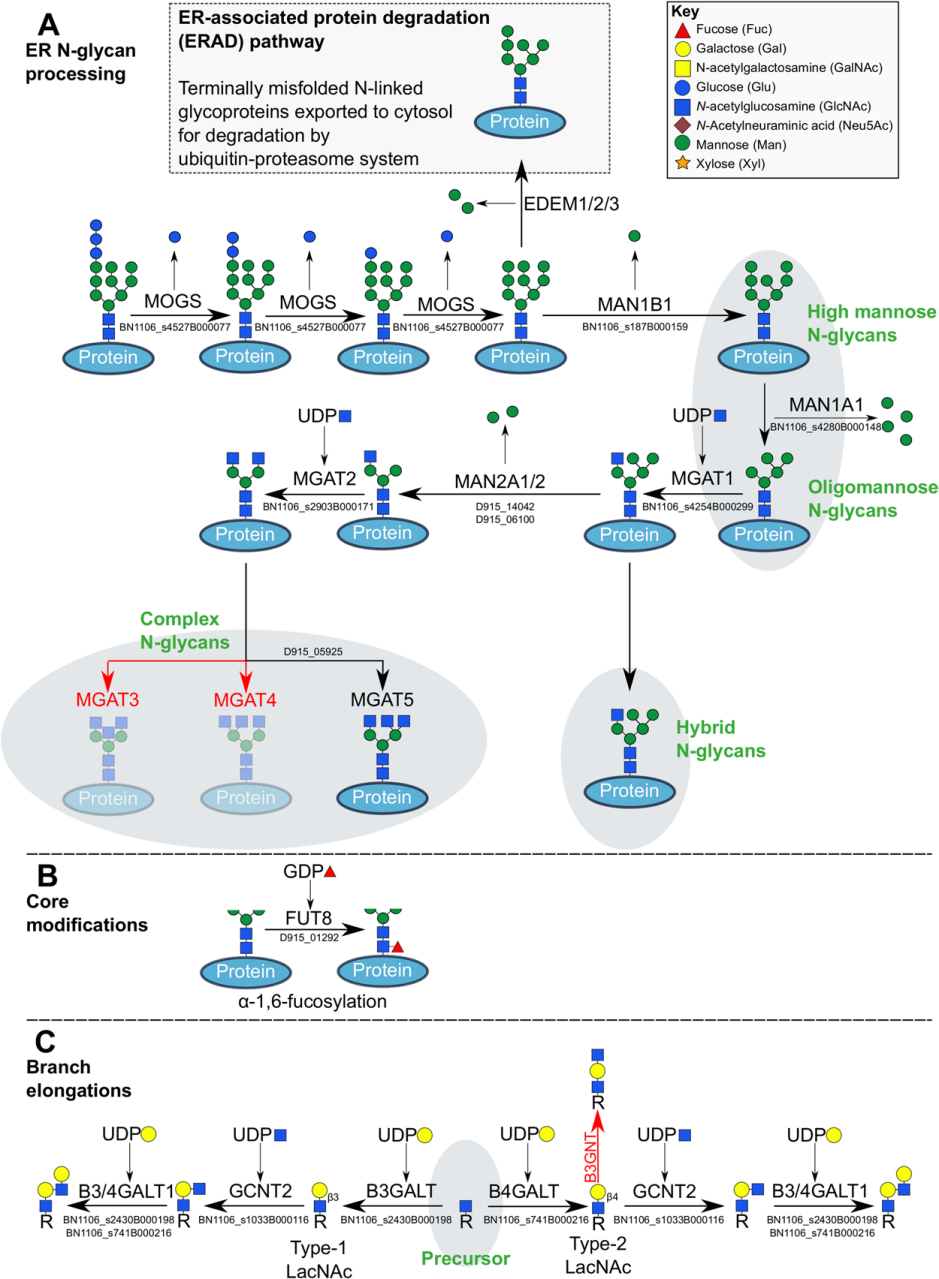
Post-synthesis processing of N-glycans, and branch extensions of N- and O-glycans. (**A**) Man_9_Glc_3_GlcNAc_2_ N-glycans are processed by a series of glycosidases towards a high-mannose or oligomannose state, after which a series of glycosidase and glycosyltransferase activities mediate conversion to either hybrid or complex N-glycans. Human gene names and *Fasciola hepatica* orthologues are indicated at each step. Red arrows and text indicate the absence of an orthologue in *F*. *hepatica*. (**B**) The N-glycan chitobiose core can be fucosylated by a FUT8 alpha-1,6-fucosyltransferase orthologue. (**C**) Glycan branches on either N- or O-linked glycans can be extended by the limited complement of glycosyltransferases that are present in the *F*. *hepatica* genome.

Following *en bloc* transfer of the Man_9_Glc_3_GlcNAc_2_ precursor glycan to an N-glycosylation site on a newly synthesised glycoprotein, N-glycans undergo extensive remodelling within the ER and Golgi. During the first phase of processing, specific glycosidases remove individual monosaccharides, converting Man_9_Glc_3_GlcNAc_2_ into Man_5_GlcNAc_2_ (Fig. 5A). These ER glycosidases form a stereotyped pathway that is conserved across Eukaryota^21^, and all are found in the *F*. *hepatica* genome (Fig. 4A, Supplementary Dataset S2). The first trimming steps involve the sequential action of mannosyl oligosaccharide glucosidase (MOGS) to remove three glucose molecules from Man_9_Glc_3_GlcNAc_2_; we identified two *F*. *hepatica* MOGS orthologues (BN1106_s4527B000076, BN1106_s4527B000077; Supplementary Dataset S2), both of which identified MOGS as a best reciprocal hit, and both of which contained glycoside hydrolase family 63 domains (IPR004888). At this point, any exposed Man_9_GlcNAc_2_ on misfolded proteins may be targeted by one of three ER degradation enhancing mannosidases (EDEM), initiating removal from the ER and destruction within the ubiquitin-proteasome system^55^; orthologues of all three exist in *F*. *hepatica* (EDEM1, BN1106_s482B000295; EDEM2, BN1106_s3059B000208; EDEM3, BN1106_s1073B000174; Supplementary Dataset S2). N-glycans on correctly folded proteins are processed by mannosidase MAN1B1 (two orthologues in *F*. *hepatica*: BN1106_s187B000154 and BN1106_s187B000159) to Man_8_GlcNAc_2_, and then are subject to variable levels of further mannosidase activity by MAN1A1 (one *F*. *hepatica* orthologue: BN1106_s4280B000148) to produce high- and/or oligo-mannose glycans bearing between five and eight mannose residues (Man_5–8_GlcNAc_2_). These N-glycans are not processed further within the ER processing pathway. Mass spectrometry confirms that such mannosidic structures comprise the bulk of tegumental and secreted N-linked glycoproteins in *F*. *hepatica*^13,14^, suggesting that these glycoproteins exit the N-glycosylation pathway at this point. Nevertheless, downstream elements of the pathway also exist in the *F*. *hepatica* genome, supporting the existence of some hybrid and complex N-glycans on *F*. *hepatica* glycoproteins.

Mannosyl glycoprotein N-acetylglucosaminyltransferase 1 (MGAT1; BN1106_s254B000299) begins the transition towards hybrid and complex N-glycans, by adding a GlcNAc onto Man_5_GlcNAc_2_ (Fig. 5A). Branch elongation can then occur on this GlcNAc residue (as described below), to form hybrid N-glycan structures. Further mannosidase activity on Man_5_GlcNAc_2_ by MAN2A1 (two orthologues: BN1106_s233B000264, D915_14142) and MAN2A2 (two orthologues: BN1106_s233B000265, D915_06100) removes two further mannose residues to produce Man_3_GlcNAc_2_, committing the glycan towards production of a complex N-glycan structure. Addition of a further GlcNAc by MGAT2 (BN1106_s2903B000171) forms the basic structure from which bi- and tri-antennary complex N-glycans can be formed. From this point on, several genes appear absent from the *F*. *hepatica* genome. In humans, three MGAT genes operate to: (i) add GlcNAc in β4 linkage to the core mannose to produce a tri-antennary glycan (MGAT3); (ii) add an additional GlcNAc to the α-1,3 arm (MGAT4); or (iii) add an additional GlcNAc to the α-1,6 arm (MGAT5). MGAT5 (four orthologues: BN1106_s243B000415, D915_05925, D915_11092, D915_01605) is the only one of these three to appear in the *F*. *hepatica* genome, suggesting that *F*. *hepatica* produces a much more limited repertoire of complex N-glycan structures than its mammalian hosts. The most complex N-glycans reported in MS analyses of *F*. *hepatica* tegumental and secreted glycoproteins were truncated hybrid structures^13,14^. Our genomic data predict that MGAT5-generated complex structures should also exist, although their absence from published MS datasets suggests either that: (i) the genes responsible for complex glycan synthesis may be spatially or developmentally regulated in tissues or life stages not yet sampled by MS studies; or (ii) these structures were expressed at levels below the sensitivity of the MS methods employed.

### O-glycans

**Figure 6:**
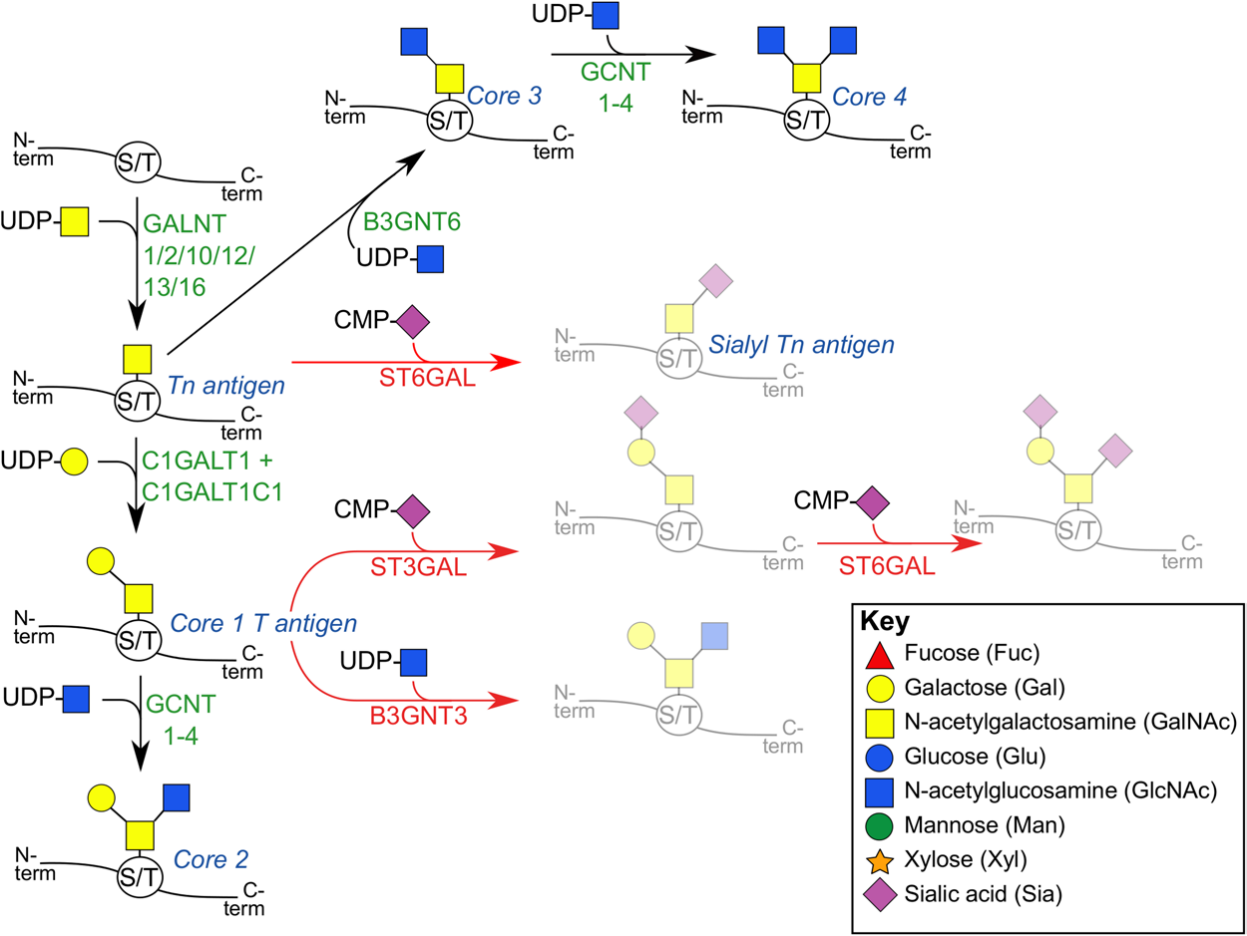
Synthesis of O-glycans in *Fasciola hepatica*. O-glycans are synthesised by the stepwise addition of sugars to Ser/Thr sites on protein, via an O-glycosidic bond. These reactions are catalysed by glycosyltransferase enzymes (human gene names in green text), to produce the structures shown (structure names in blue text). Common O-glycan structures that cannot be produced by *F*. *hepatica* appear faint grey, with enzymes not found to be present shown in red text. Full orthologue data are shown in Supplementary Dataset S2.

O-glycans are synthesised in the Golgi by sequentially acting glycosyltransferase enzymes, where the common first step is the addition of GalNAc to serine or threonine, catalysed by polypeptide N-acetylgalactosaminyltransferase (GALNT), to form a structure called the Tn antigen (Fig. 6). O-glycoproteins are abundant in secreted mucus, and are therefore also termed mucins^56^. In *F*. *hepatica*, mucins are secreted during metacercarial encystment, to form a mucopolysaccharide layer of the metacercarial cyst wall^57^. Mucins have also been identified in adult liver fluke, including in the tegument^12^, where they may form a protective or lubricating layer of the parasite surface, and/or mediate interactions with the host immune system. Previous studies show that the platyhelminth O-glycome is dominated by structurally simple O-linked polysaccharides, which generally lack the complex extended chains seen in mammalian glycans^12,58^. However, longer O-glycan chains consisting of up to nine monosaccharides have been reported in cestodes^50,59^, and schistosomes^42,43,60^.

The human genome contains 18 GALNT genes (GALNT1-GALNT18), and the *F*. *hepatica* genome contains orthologues of several of these: GALNT1 (BN1106_s1253B000118, D915_04596, D915_04597, D915_11750, D915_00062), GALNT2 (BN1106_s5930B000044), GALNT10 (BN1106_s1334B000257), GALNT12 (BN1106_s8167B000031), GALNT13 (BN1106_s2029B000103, BN1106_s2104B000156, BN1106_s2104B000157, BN1106_s2625B00067, BN1106_s2625B00068), and GALNT16 (BN1106_s2029B000102, D915_08493, D915_08494). The Tn antigen can be converted directly into either Core 1 or Core 3 T antigens. Core 1 synthesis is performed by addition of Gal by C1GALT1 (thirteen orthologues of which exist in *F*. *hepatica*, Fig. 6; Supplementary Dataset S2), operating alongside a molecular chaperone (C1GALT1C1, D915_14130); Core 3 synthesis is catalysed by B3GNT6 (D915_10349), which adds a GlcNAc to Tn antigen (Fig. 6). Core 1 and Core 3 structures can both be converted to, respectively, Core 2 and Core 4 structures, by GCNT3 (six orthologues: BN1106_s4397B000042, BN1106_s5966B000046, BN1106_s773B000385, BN1106_s933B000129, D915_12022, D915_15676; Fig. 6). These data support the likely existence of conserved Core structures in the *F*. *hepatica* O-glycome. While the existence of Tn structures is confirmed by immunochemical analyses of the adult tegument^11,12^, further analysis of the *F*. *hepatica* O-glycome is needed to confirm our predictions.

### Glycan chain extension

The core N- and O-glycan structures shown in Figs 5 and 6 can all be subject to further addition of monosaccharides into linear or branched chains, greatly increasing the complexity of the final structures produced by both N- and O-glycosylating pathways. In one of the simplest modifications, mannosidic, hybrid and complex N-glycans may be decorated by the Man_3_GlcNAc_2_ glycan stem (i.e. the chitobiose core) closest to the protein. Here, fucose may be added to the chitobiose core in α-1,3 or α-1,6 linkage, GlcNAc in α-1,6 linkage, or xylose in β1,2 linkage to a core mannose^21^. Our dataset contained only genes associated with α-1,6 fucosylation, catalysed by α-1,6-fucosyltransferase (FUT8) orthologues (D915_01291, D915_01292) (Fig. 5B). We did not identify orthologues of FUT1-FUT7, suggesting that fucosylation of terminal residues may not occur in *F*. *hepatica*. This is interesting from an immunological perspective, since Le-X/LDN motifs are abundant and immunogenic on glycoproteins of other trematodes such as *Schistosoma spp*.^42,43,60^. Our prediction of the absence of these motifs from *F*. *hepatica* glycoproteins is supported by their absence from *F*. *hepatica* glycomic datasets^13,14^.

Both hybrid and complex N-glycan branches terminating in GlcNAc can be subject to branch elongation, because GlcNAc forms a platform onto which further monosaccharides can be added (Fig. 5C). In many organisms, these chains can consist of galactose, GalNAc, GlcNAc and sialic acid in linear or branched chains, constructed by sugar-specific glycosyltransferases. These chains may then be capped with sulphate and/or phosphate residues, mediated respectively by sulfotransferases and phosphotransferases. We discovered that the *F*. *hepatica* genome contains a very limited set of glycosyltransferases. Beta-1,4-galactosyltransferases (B4GALT1-7) catalyse the transfer of galactose to terminal GlcNAc. We identified orthologues of B4GALT1 (BN1106_s741B000216, D915_08355), B4GALT2 (BN1106_s5191B000044, BN1106_s741B000214), B4GALT4 (BN1106_s547B000277), B4GALT5 (BN1106_s5562B000030, D915_02463), B4GALT6 (BN1106_s1105B000131), and B4GALT7 (BN1106_s1573B000174) (Fig. 4C). The β4-linked GlcNAc-Gal unit produced by this transferase is the basic unit of a “type 2” version of an N-acetyllactosamine unit. The type 1 version of this structure is produced when the sugars are in β3 linkage, a reaction catalysed by beta-1,3-galactosyltransferases (B3GALT1: BN1106_s2430B000198, BN1106_s4244B000584, D915_14141; B3GALT5: BN1106_s1989B000317, BN1106_s2686B). Type 2 (but not type 1) N-acetyllactosamine can be further supplemented by addition of GlcNAc to the terminal galactose by beta-1,3-GlcNAc-transferase (B3GNT2-B3GNT7). *F*. *hepatica* lacks orthologues of the B3GNT variants that are known to have activity against glycoproteins (B3GNT2-B3GNT4) but does possess orthologues of B3GNT5 (D915_05233, D915_06123, D915_06124, D915_11823) and B3GNT7 (D915_10349) which in humans transfer GlcNAc onto lactosylceramide and keratane, respectively. If the assumption of direct functional conservation between orthologues is correct, it would indicate that more complex polysaccharide extensions are absent from *F*. *hepatica* glycoproteins. This hypothesis is strengthened by the absence of extended hybrid/complex-glycan chains from MS-based descriptions of *F*. *hepatica* N-glycans^13,14^. If glycoprotein-focused B3GNT activity does exist in *F*. *hepatica*, the resulting terminal GlcNAc could subsequently receive galactose via B4GALT activity as described above, to produce chains of repeating poly-N-acetyllactosamine subunits. Available data show no evidence that *F*. *hepatica* can produce such structures on N-glycoproteins^13,14^, but no studies have yet addressed their existence on O-glycoproteins or glycolipids.

Branching of LacNAc (Type 1 and Type 2) is catalysed by N-acetyllactosaminide β-1,6-N-acetylglucosaminyl transferase (GCNT2: BN1106_s1033B000116, D915_12022, BN1106_s9586B000050, BN1106_s12390B000006, D915_13029), which adds GlcNAc in β1-6 linkage to galactose (Fig. 5C)^61^. This would permit further repetition of the chains described above on these branched structures. Alternatively, β-1,4-N acetylgalactosaminyltransferase 1 (B4GALNT1) can catalyse the addition of GalNAc onto GlcNAc to produce a so-called LacdiNAc motif. We did not detect B4GALNT1 orhtologues, designating B3GALT, GCNT2 and B4GALT1 as the only branch extension enzymes that we detected in the *F*. *hepatica* genome. This genomic simplicity is consistent with the hypothesis that the protein-linked glycans of *F*. *hepatica* are structurally simple; MS datasets support this hypothesis, by showing that N-glycans on *F*. *hepatica* tegumental extracts and secreted cathepsins (B3 and L3) consist solely of oligomannose or truncated hybrid/complex glycans^13,14^. By focusing on the tegmental and MS datasets, these MS studies describe valuable data on the glycomic configuration of the host-parasite interface. MS methods have not yet been used to describe the glycan composition of *F*. *hepatica* internal cells/tissues. The absence of enzymes capable of creating more complex N-glycans in our genomic data suggests that internal N-glycans exhibit similarly low complexity as secreted/external N-glycans. Although MS datasets show the presence of sulphate on N-glycans, we did not identify any sulfotransferase orthologues in the *F*. *hepatica* genome. Humans possess 15 carbohydrate sulfotransferase (CHST) genes, of which CHST7-CHST9 act on N- or O-glycan substrates; none of these were present in *F*. *hepatica*, suggesting either that they were absent from current predicted protein datasets, or that *F*. *hepatica* sulfotransferase orthologues have diverged such that our methods cannot detect them.

### Gene and protein expression

**Figure 7:**
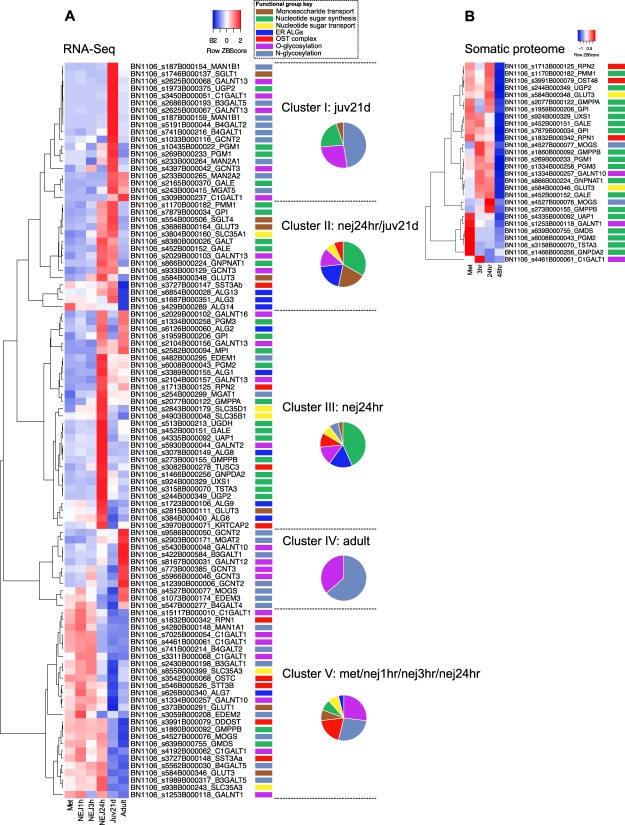
Developmental expression of 101 protein glycosylation genes in *F*. *hepatica* RNA-Seq and proteomic datasets. (**A**) Heatmap illustrates changes in RNA-Seq RPKM (Reads Per Kilobase of exon model per Million mapped reads) per gene across datasets (red = highest RPKM, blue = lowest RPKM) representing metacercariae (met), nej1hr (NEJ 1hr post excystment), nej3hr (NEJ 3hr post excystment), nej24h (NEJ 24h post excystment), juv21d (liver stage juveniles 21 days post rat infection), and adult parasites. Hierarchical clustering identified five expression clusters based on predominant expression of genes in specific life stages as indicated. Pie charts indicate the proportion of functional groups appearing in each expression cluster. (**B**) Heatmap illustrating protein abundance (expressed as exponentially-modified protein abundance index; emPAI^64^ from proteomic datasets representing the somatic proteomes of metacercariae, 3 h, 24 h and 48 h NEJs/juveniles). Individual genes are indicated by *F*. *hepatica* genome sequence ID (WormBase ParaSite genome PRJEB6687), and that sequence’s closest human orthologue gene name. Each sequence ID is associated with a colour code, identifying its functional group, as indicated in the key.

In developmentally staged transcriptomes from intra-mammalian parasites^8,9^, we detected 101 glycogene transcripts (66% of total; Fig. 7, Supplementary Dataset S2). Every gene with an annotated gene model in the PRJEB6687 genome^8^ was present in the RNA-Seq dataset. Reflecting the numbers of genes in each functional group (Supplementary Dataset S2), the data were dominated by transcripts related to N-glycan processing (25% of all expressed genes), nucleotide sugar synthesis (24%) and O-glycosylation (22%) related genes. ER ALG and OST transcripts accounted for 9% each, sugar transporters 7% and nucleotide sugar transporters 5% (Fig. 7). Hierarchical clustering supported the existence of five major expression clusters. These clusters defined distinct developmental expression patterns, describing genes expressed most highly in 21-day juveniles (Cluster I, 19 genes), 24 h NEJs and 21-day juveniles (Cluster II, 15 genes), 24 h NEJs (Cluster III, 30 genes), adults (Cluster IV, eleven genes), metacercariae, 1 h, 3 h and 24 h NEJs (Cluster V, 26 genes). Each cluster contained genes from several of our functional groupings, but groupings were not equally represented between life stages, suggesting developmental regulation of groups of disparate genes between life stages. Such regulation is consistent with fine control of protein glycosylation patterns that change as parasites progress through intra-mammalian development.

Exploring our previously published proteomic datasets for peptides relating to our glycosylation genes uncovered 29 genes represented by at least 2 unique peptides in one or more of these datasets (Supplementary Dataset S5)^9,14,62^. Notably, these datasets identified three glycosylating proteins within secreted proteome datasets. A phosphoglucose isomerase (PGI) orthologue (BN1106_s1959B000206) represented by three unique peptides in an adult tegumental extract (FhTeg), was also represented across *in vitro* collected (after 1 h, 3 h and 24 h incubations) excretory/secretory (ES) proteomes from NEJs, implying that this protein may be derived from the NEJ tegument. This PGI orthologue was not detected in proteomes relating to extracellular vesicles (EVs) collected from Adult parasites, suggesting PGI may be released by sloughing during normal turnover of the tegument. Adult worm EV proteomes did contain peptides derived from GALNT10 (BN1106_s1334B000257) and B4GALT2 (BN1106_s741B000214), identifying these peptides as derived from the EV lumen. The release of EVs by *F*. *hepatica* has been reported^62^; the presence of glycosyl-transferase factors within EVs correlates with the established glycosidase activity in *F*. *hepatica* ES products^63^, and presents an intriguing avenue for future research on the host-parasite interface. Notably, both *F*. *hepatica* GALNT10 and B4GALT2 proteins are annotated with metal-binding proteins by InterProScan, suggesting that these secreted proteins may have roles in scavenging metals from the host environment. Binding of metal ions within host cells may be a means for blocking metal-dependent enzyme reactions or interfering with host metal ion homeostasis. Somatic proteomes from metacercariae, 3 h, 24 h and 48 h NEJ/juveniles contained peptides derived from 28 genes (Fig. 7B). These were overwhelmingly derived from genes associated with nucleotide sugar synthesis (65% of total), indicating the high levels of protein expression from these essential genes, and reinforcing the independence of *F*. *hepatica* from its host in terms of glycan synthesis.

## Conclusions

This work has exploited recently available genome data from *Fasciola hepatica* to provide the most detailed description of protein glycosylating genes available for any parasitic helminth. We have identified 150 distinct *F*. *hepatica* genes associated with sugar acquisition, nucleotide sugar synthesis, nucleotide sugar transport, N-glycosylation and O-glycosylation. This complement was characterised by extensive gene duplication, with 31 genes represented by multiple paralogues. Without these paralogous duplicates, the *F*. *hepatica* complement contained just 87 orthologues of 153 human glycosylating genes. When analysed in terms of the known functions of their human orthologues, the *F*. *hepatica* glycosylating genes explain the structural simplicity of the overwhelmingly mannosidic N-glycans known to be present on tegumental and secreted glycoproteins. The absence of genes associated with complex structural modifications predicts pervasive structural simplicity of liver fluke glycans across tissues and life cycle stages. Some genes were differentially expressed across developmentally-staged RNA-Seq and proteomic datasets, suggesting a level of developmental regulation of protein glycosylation profiles. This work provides a foundation for future functional genomic interrogation of liver fluke protein glycosylation, which might support the development of new fluke control methods.

## Electronic supplementary material

S1

S2

S3

S4

S5
