# Supplementary material for: In silico analyses of protein glycosylating genes in the helminth Fasciola hepatica (liver fluke) predict protein-linked glycan simplicity and reveal temporally-dynamic expression profiles
Source: Sci Rep. 2018 Aug 3;8:11700. doi: 10.1038/s41598-018-29673-3 (PMC6076252; doi:10.1038/s41598-018-29673-3)
Supplement: Supplementary file 1 — S1 [file 41598_2018_29673_MOESM1_ESM.pdf]

In silico analyses of protein glycosylating genes in the helminth Fasciola hepatica (liver fluke) predict protein-linked glycan simplicity and reveal temporally-dynamic expression profiles

Paul McVeigh, Krystyna Cwiklinski, Andres Garcia-Campos, Grace Mulcahy, Sandra M O'Neill, Aaron G Maule, John P Dalton

Supplementary Dataset S1. Human query genes used for BLASTp searches.

ER precursor N-glycan (Man9Glc3GlcNAc2) synthesis
ALG1
ALG2
ALG3
ALG6
ALG7
ALG8
ALG9
ALG10
ALG11
ALG12
ALG13
ALG14

N-glycan processing
EDEM1
EDEM2
EDEM3
MOGS
MAN1A1
MAN1A2
MAN1B1
MAN1C1
MAN2A1
MAN2A2
MGAT1
MGAT2
MGAT4A
MGAT4B
MGAT5A
MGAT5
MGAT5B
FUT1
FUT2
FUT3
FUT4
FUT5
FUT6
FUT7
FUT8
FUT9
FUT10
FUT11
XYLT
XYLT2
B3GALNT2
B3GALT1
B3GALT2
B3GALT3/B3GALNT1
B3GALT4
B3GALT5
B3GALT6

B3GNT1/B4GAT1
B3GNT2
B3GNT3
B3GNT4
GCNT2
B4GALT1
B4GALT2
B4GALT4
B4GALT5
B4GALT6
B4GALT7
B4GALNT1
B4GALNT2
B4GALNT3
B4GALNT4
ABO
A3GALT2
CHST1
CHST10
CHST11
CHST12
CHST13
CHST15
CHST2
CHST3
CHST4
CHST5
CHST6
CHST7
CHST8
CHST9
CHST14
GNPTAB
ST3GAL1
ST3GAL2
ST3GAL3
ST3GAL4
ST3GAL5
ST3GAL6
ST6GALNAC1
ST6GALNAC2
ST6GALNAC3
ST6GALNAC4
ST6GALNAC5
ST6GALNAC6
ST8SIA1
ST8SIA2
ST8SIA3
ST8SIA4
ST8SIA5
ST8SIA6
GAL3ST1
GAL3ST2
GAL3ST3

```
GAL3ST4

# Nucleotide sugar synthesis
CMAS
GALE
GALK1
GALT
GCK
GMDS
GMPPA
GMPPB
GNE
GNPD2
GNPNAT1
GPI
MPI
NANP
NANS
PGM1
PGM2
PGM3
PMM1
TSTA3
UAP1
UGDH
UGP2
UXS1

# O-glycan synthesis
GALNT1
GALNT2
GALNT3
GALNT4
GALNT5
GALNT6
GALNT7
GALNT8
GALNT9
GALNT10
GALNT11
GALNT12
GALNT13
GALNT14
GALNT15
GALNT16
GALNT17
GALNT18
C1GALT1
C1GALT1C1
GCNT3
GCNT6
GCNT7
B3GNT6
ST6GAL1
```

ST6GAL2
GAL3ST4

Oligosaccharyltransferase complex
DAD1
DDOST/OST48
KRTCAP2
MAGT1
MLEC
OST4
OSTC
RPN1
RPN2
SST3A
STT3B
TUSC3

Sugar uptake/transport
GLUT1
GLUT2
GLUT3
GLUT4
GLUT5
GLUT6
GLUT7
GLUT8
GLUT9
GLUT10
GLUT11
GLUT12
GLUT14
HMIT
SGLT1
SGLT2
SGLT4
SGLT5

Nucleotide sugar transport
SLC35B4
SLC35C1
SLC35A2
SLC35D2
SLC35D1
SLC35A1
