# Supplementary material for: In silico analyses of protein glycosylating genes in the helminth Fasciola hepatica (liver fluke) predict protein-linked glycan simplicity and reveal temporally-dynamic expression profiles
Source: Sci Rep. 2018 Aug 3;8:11700. doi: 10.1038/s41598-018-29673-3 (PMC6076252; doi:10.1038/s41598-018-29673-3)
Supplement: Supplementary file 3 — S3 [file 41598_2018_29673_MOESM3_ESM.pdf]

In silico analyses of protein glycosylating genes in the helminth Fasciola hepatica (liver fluke) predict protein-linked glycan simplicity and reveal temporally-dynamic expression profiles

Paul McVeigh, Krystyna Cwiklinski, Andres Garcia-Campos, Grace Mulcahy, Sandra M O’Neill, Aaron G Maule, John P Dalton

Supplementary Data S3. Fasciola hepatica protein

>BN1106_s187B000159
MQVRSGKILRRLHSLPTYHGLLPNFIDREGGPNWDSRIGIGAHGDSYYEYLLKVWIQTGK
KINALRDDYRKAIKGIQDHLVGYSKPNNLMFIGELNDGDFTPLMDHLVCFLPGTLILGYR
NGMPRSHMKMAEQLLETCFQLYNQTETGLSPEIARYSTDVRSKKDFTPEVCIPFLMI*
>BN1106_s1334B000257
MRIRPTRIIRLIAYVATFFFSSSIIFYALTPSKHQSSNQEAVLASFLLQPRASLPFVSGV
SNNELVSWEDENLKETEAKREGPGEQGRPVRLVGPEKKISEKFFNQNGFNIYISDKIAVD
RAVGDIRHPRCKSMSYLRKLPTASIIVPFFEEHWSTLLRTFVGVLKRTPNELIKEVILVD
DGSTLRPELKDKLDNYLKTNYPDGLVRVIHSPSREGLIRARITGAKAATGDVLVFLDSHC
EPSHNWLPPLLDPIARDYRTVVCPFIDVIDADTFEYRAQDEGARGAFDWELYYKRLPKLP
KDEPSPELPFDSPVMAGGLFAISSKWFWELGGYDPGLEIWGGEQYELSFKLWMCGGRMID
VPCSRVGHIYRKHPTNFPSARGKGDFVGRNYKRVAEVWMDEYKEYIYNRRPHYRALDTGD
LSEQHAVRERLKCKSFKWFMTEVAFDLTKKYPLIDPIPAATGDIRSVVEPSLCIEALEAS
EVKPIRLSKCVRDGAGLNGMQKFELSYHDDIRPVKL*
>BN1106_s15117B000010
MIEFVTPMKGLSVHFADGSRRMFSLPFGFAVGFCLSAYFSPFYWNRGSQALTCQQTLLRP
LHADISLTRNSGGPSILCWITVMPLNHERKAKHIKATWAKRCDKYIFMSSINDPDLPSIA
AVQFESRDMLWNKTHFALTYISKHFGEKFDYFFKADDDT*
>BN1106_s1973B000375
MTGVPTDTTFQFLSTKSSFREVPFSDGENNIRAALTELRSSMSDEERENYPIDEETFLRM
YRAYLKKDDQVLHWSDITQPEDLIGQYETLAEPNHSEAVSLLNKLVVIKLNGGLGTSMGC
TGPKSLIPVRDGKNFIDLTVEQITELNVEYGCDVPLVLMNSFNTNSETLQALNKSGENRP
QIFTFEQNRFPRLSPETGLPLKSVGTFPVGSVLWYPPGHGDVYRSFEKSGLLEKFMKEGK
TWVFISNIDNLGATVDTAILNYLENSHDQDHGCDFLMEVTPKTPSDIKGGTLVKFGRKLR
LLELAQVPENYIEDFTSVRKFKFFNTNNLWVNLKAMAKLLVEKRISMELIVNPKTVNKSI
PVLQLEEAAGAAIHNFDKPRGLTVPRSRFLPVKLTADLLLVMSNLYILEKGRIVPSPLRN
FPTLPLVKLGKHFSNIKDFRERLKSIPDMLELDHLTVSGDVHFDKNVTLKNNGTCRGSDG
IRFNPESADNVLVCAAEYHTVVEEVSLEIGQKRSREPEFWG*
>BN1106_s2430B000198
MAEKNERQLFSSDSSKGARRKLNQSDFKVKPLVSTGSEDLHFPLDLDFPELVRKVLANET
VPVKPINRPDFPILVPLADKCQPSSLLFKLRPDLLVLIKSAPSHFALRDAIRIGWGDEKC
WGGRQIVRLFLLGTVPANDSNTAARLEMEMEMYSDIIQQEFIDHYYNNTYKIMFGLEWAI
KYCANAPLIMFVDDDFFVYPKNVIAYIEGLSVGIQDRLISGYVWSNAIPVRANRSFDGKW
FVSKSEFPDAHYPPYVAAGHFFLSQKMARELYVASQYTRYLRFDDVFLGIILKKLVRVPI
HLKQIHAYWAIYQNSTAFQTIISSHRFGDPVLQLKAWDHLNCSQFCTNL*
>BN1106_s1687B000351
MVLKAAISLSKSIVFTERGYMPVAFILVAAELLLCSLIVEKVKYTEIDWVAYMQEVEHFL
NGTLDYDQIKGQTGPCVSRGGLTRFLSLHKPKCTARPDAASVLYPLFVCNFVGIAFSRSL
HYQFYVWYFHTLLYLLWSNSQLPTPVRFLLLGLIELCWNTYPSTVYSSGLLHACHGALLI
CLLWSAPDGCEELRRPNTQSAKQYAKPTPSKANHKRNKPKQA*
>BN1106_s1170B000182
MSAGSQFSIVDSIVKGKFVSAAMDSTQGVICLFDVDGTLTKPRNVIKPEFLQQLLALQSR
IPIAVVSGSDFPKVAWQLGGEPVMEKFGYVFSENGLVVHRFGKMIQSTSIVDHLGEELIQ
KFINYALGYMSKLELPRKRGTFVEFRKGLINICPIGRSCTQAERDEFAEYDDIHRIREHF
VEDMKKKFGHTDLQFAIGGQISIDVFPKGWDKRYCLQFLSEFPKIHFFGDRTTEVGF*
>BN1106_s1334B000258
MATASIRNAVAAALKEHKPTSGVSFGTAGFRCQASKLSGIAFRVGVLAAVRSLNKGQFVG
VMITASHNPPGDNGIKLIDPDGGMLKASWEPVVMEFMECSESDGSTWLAGHLNNPETESI
PRVLVGFDTRDSSPQLAAEVRQGVEAMHGFCLCLNLVTTPQLHYAVYHMGVRQNSENPGR
PLAPAISAWITAERALLSVHT*
>BN1106_s1253B000118
MKVIFLRWRRLVPLLILLACFVLLFTQWNYKLEFDVDKQRGLGKPELPHANRAPVAPKDI

KDHPVLPQVHQPDAIDAMKKHEALNPAAVNPVVQGKQSVIEEFNLYPRVPPPKSDENSVG
PGERGAGFTVNRDRLSQQEQARYDQGWQDNAFNQYVSDLISVRRYVVDQREGECLTKKYQ
ETLPSTSVIICFHNEAWSVLLRSVHSVIDNSPPALLKEIVLVDDFSDKPHLKEPLEEYMA
QLKIVKIVRAKQREGLIRARMLGARASSAEVLTFLDSHIECTKGWLEPLLDRIQTDKTNV
VVPVIEVIGDSDLKYHRANVQSIQVGGFDWDLIFHWHPPPERDRKRPGAPYSPIRTPTMA
GGLFSISREFFKNLGYYDEGMEVWGGENLELSFKTWMCGGTLETIPCSHVGHIFRKRSPY
KWESKFTSPLRRNTVRLAEVWLDDYKRFYYAKIGFQLGDYGDVSDRKQIRQRLQCKPFKW
YLENIYPELFVPSNAIASGDIENVESAHCIDAPLKSKDDRTVIVGMWPCHRQGGNQFWMM
SKNGEIRRDSKCWDVGTNPARISIIDCHGGRGNQEFAYLESGEIKHGERCLEMSLDKQTL
QVAFCTGSKRQQWKFNRKPFMPPESQ*
>BN1106_s2029B000103
MIFWTTNVQLHLPPTTHTYSDLLCFLSQIESYSDSVCLDAPTDAKDQSNVVRTLRCHRQR
GNQFWLLSEAGEIRRDDRCFDSGVRPKSLGLFDCHGLGGNQRFTYEQDETIRHGDQCVEY
DGLDKPIVFKPCDGSVRQKWKFNRKPYKPKISASSS*
>BN1106_s1959B000206
MSALKEFLSSFKKVPCTMKEMFQSNPNRFNEFSHVLKLQHGSILLDFSKNLIDTDTFARL
IKLARDSNVERMRDAMFSGEKINFTENRAVLHVALRNRSNTPILVDGKDVMPGVNAVLRH
MKEFCDSVRSGEWKGYTGKAITDVINIGIGGSDLGPVMATECLRPYAKHIKVHFVSNIDG
THISETLKKLNPETALFIVASK*
>BN1106_s1746B000137
MSYIIGVLDGADIGVIVGYFAVVIGVGVWSSCCNRGSVGGYFLAGRSMNWALVGASLFAS
NIGSGHFIGLAGSGAASGIAVGVFELSADLYAGAIFIDQALGLNLYIAIILLLIISGLFT
ILAFIHVGGFESMVTKYFNSIPNTTRVHQLTEATTDPNNLTLPSTVHDNYLKCGVPNEDA
FHMFREVNDPNLPWTGVIFGLTISNIWYWCTDQVRICESEIEAQLVVQVIVQRTLSAKNL
THAKGGCLLAGVLKLLPLWLLVFPGMIARILFVDEVGCADPDVCRQVCGKERGCTDIAYT
RLVLRLLPSGARGLMLAVMIASLVSSLTSIFNSSSTLFTMDIWRRFRPRARSAELMVVGR
VSTLALIGISVAWIPIVQSSGELFHYIQSVTSYLAPPVCGVFLLAVFWPRFNENGAFYAL
IVGLAVGIIRFVWEAVYSRLACGEVQEETIAQKMVTKLHYLHFGIILLFISCSVGVIVSL
LTKPLPEKYTLGMTYWTRNYKPSEVKRTSDEVNDHQYGVEETEDPACIHAFVDVEP
>BN1106_s2104B000156
MKFRKTLTVKLILIVCALWLLLQVLVYEVSSVDDSRNTNWFHRLTGYFHSSNVPSIALPK
FVPGIRSQGHGYGHLGQAVQLSASLYAESRRSFSLHQFNLVASDLIGPNRTLPDHRKPDC
QNIPVTNDLIALRTSVIIVFHNEALSTLLRTVNSVIQTSPHELVAEIVLVDDASTLADAD
GTECWRYWEYHIRDRQF*
>BN1106_s233B000264
MLLNLTLFTGDLFTYADRDHDYWSGFFTSRPVEKFLTRTLESELRSSELLYTYARHLIQR
LPDSSLNETVHLLDDRITLARRALGLFQHHDGVTGTAKSHVVADYNRRLRSALNDCRLIS
AVSSAALLLALPSPAADLTKRAVNPQQVINTIREVHRLPKESQGVATIISMEDLYFREAA
PVPYRIKIETTHESIPIVIFNPLLQPRITTVTVELIGVYEHFNVKFAPHVSESMENPITM
QIESPDSDAGQQTRLRIGPVHLAPLSLSQLVIEHSETHSERSVIVSYEPPVRTQSSNLIW
LNSDSIGLGFDAHTGLLRYLIDSRLNVVLNVTIDFLIYQTNDKAESRSGAYLFIPNSPGR
VMELPNKPKVRITRGPLVQEIILYTPLVQHSVRLYKYPSNVIEIENTVSLGKFHPQNVEL
VMRIQSNVSNSDRTFFTDSNCFQVCLIMVEHNFPVYHPLSDTFIRCHRSTPAGIMHIHPC
EATPLLSTEAHAVVNDLIRPIQRFLVGHNISSFMARTVHLMPNFTMPKDYDLVSLKTFHH
NTELFKTFSDYYGAQIGMLLRRLPVGFGEQIPHEISVAQMFSRLNRTRAVRTPLTLIPEM
SSVRSLQGRLPTVTVKPMELEAYLLTV*
>BN1106_s12390B000006
MWQRSKLPIHLSCSRVLLVFSVLLIGGCVLFLYLRFDINTDTTDHWSEYGLITNATLLYL
PTVLTRKDNLCPIVLENQTNMLFADTLVNLVRSDTGNFIPSDDQKCREFKQIHEDHVKVS
DEELEFPLAFSFNVHKEFNQFARLFRAVYRHHNAXXXXXXXXXXXXXXRKVEYLATCFGP
NVHVIPLRQSISIRWGDLGTLEAWIRCADFFLKQSVIRWKYMLNGSGQEFPLRTNWELVK
ALKAINGSNIVESDYPNTGKPRVPSEPLSFNLNQWISKHTPIHCYKEKCS*
>BN1106_s2104B000157
MRDAVSSLGVARVQSAKTERKLGEPELNDLHTKLESYVSKLPIHVRIERMPIRSGLVRAR

LRGAANATGKTLTFLDAHCETTVGWLEPLLAEIAVDRRRVVCPIIDVLDFETFHYSEVTS
RIWSFLLGLLRSGSDRIYGTFDWQLTFHWSPISSSESKRVGTNHSIPIRTPTMAGGLFTI
ETDYFHELGTYDTGMVVWGGENVEMSLRIWQCGGELYIITCSRVGHVFRKVSPYKWPGGV
NHVLTRNSMRTALVWMDEYQEFYLRFNPDASKADYGDVSERQALRKNLDCKSFRWYLENI
YVDSLFPLDPIALGEFKNQGSGLCLDTLGRKIDESSIGVANCHGRGGNQLFVWTGKGEIQ
SAVGCMAPDPSQSTFRFLHCRRRTNTDQVFEYRDKKLIHVKSGLCLRTKNMKELVSAPCV
EGPDFYWTLPPPFSPSMS*
>BN1106_s2029B000102
MCGGTLETVVCSHIGHLFRSRSPYKWVSNFTNPLRRNSIRLAEVWMDDYRGFYYEQFNNE
LGDFGNVSDRKAIREKLKCHDFQWYLDNVYPELFLPSRALASGDVSSFDFLMHIL*
>BN1106_s1033B000116
MIRNTLFLNVLFSCLFCLCYLLIRSFFTSVPKEQDPLSYQKTEVYNNLDRLHSAYLIQEE
ELKFPLAFSLIVYRDIDRAIRLLRTIYRPHNYYCIHVDKKASASYFLVMKEAVRRIGSNV
FLIPESERIPIVWGQMSTLDADLTCAKRLLEFSSSWRYWINLTGHEFPLRTNWELVEALK
AVRGANLVSGFRDLREFGRLPPPAITPDGVHWYKGAVHVVVRREFVDYIFKSPLAHKLLS
SLRQWEHYRRYRVFADEQYFSTLNNNPHVFNIPGSYTGNKTANGKLEFDVDLNNLSIIRH
KVWSVNVSMCGTNYWVRSICMLGMRDLKTLKKSPSLFANKFIPAVEPEGYDQLEKWIARK
VEYERINSRLHPSFDVSVYAKLDETDNHM*
>BN1106_s2165B000370
MEFLHVYRFHMVDNIKSRVLVTGGSGYVGSHTVAELINSGHSVIVLDNLVNSKAACIKRI
ERMYDCHISFYTGDLLDRSSVEAVFAKEDVDSIIHFAALKSVGASVCEPIRYYENNTMGL
INLICDLYKADSAWNIVSLRYFNPIGAHPSAQLGEDPNDRSDNLMPRISQVAGGRLPHVN
VYGSDYNTPDGTGKYKCDYQGSSVCFTVVHVWVLPVCLMQRTAYNIGTGKGYSVLEVIHA
MEKASGKPITYQLHPRRRGDTDAIYADPTLANKELGWKALYGLDKMCEDQWRWQCQNPHG
YGDEFKSGDH*
>BN1106_s1466B000256
MRLLILDTPKDVADWAAKYVMKRIIDFAPCEERYFVLGLPTGSTPLPMYKRLVEFHKAGK
LSFRYVKTFNMDEYVGLPRDHPESYHYYMYHNFFKNAHMLDGTADNLDLECERYEEEIER
AGGIDLFVGGIGPDGHIAFNEPGSSLVSRTRLKTLAKETIVANARFFNDDLSQVPERCLT
VGVGTVMDAREVMILVTGTGKALALSKAIEEGVCYLISPKYKLTGSVLISTTVVGIFPLI
LTCFR*
>BN1106_s1860B000092
MQLNTGGINLDKSGSEAEDPTAKVGNGCRIGPNVTIGAHVVIEDGVRIQNSAIFSKSVIR
SHAWLNNCIVGWRSTVGRWVRMENTTVLGENVRVKDELFLNGALVLPHNAISDSVTEPHI
IM*
>BN1106_s243B000415
MRFFVLLFMIHPPRVHLLCFPTFYPYAFLVLFRNSAHTGGPLGELVQWTDLLAGLYILGH
NVSISIEPLKLFEHFNFTPSKKPECQTEQNVFDLLFTDIVGYRRLKRLGIRIPKCKYRIL
DSFGTEALFNRNDKNSTWGGLQLNLKQFYTMFHVLDLSLVLFASFQTAHSPDNTFLGFVV
ESPMNTVTPDVRITTDETVRSKSGKPIALIYGKEAYMWKDALPYLTVLNESLELHANVMD
AALGQQFSFVVPHKCNYGTEFITLMRSAKVFVGLGFPYEGPAPLEAIANGVVFFNPLFRT
PHGRNNTKFFADKPTSRKLASQQPYLEQNVGEPYTYAIQMDKPDQIRQSVQRLLNQTAVS
LYKGCENYPKQNNANSKLTDAVRQEAVLENWVMEGQISGLD*
>BN1106_s1073B000174
MCAMRFLPLIFISAYLWVELVSMSIDDKRIMRKKVKEMFMHGYKSYRDHAYPADELMPLS
CRGRHRYTHVSRGDVDDALGNFSLTMIDALDSLFLLGELDEFESAVSLVVRDVSFDSDVD
VSVFETNIRVLGGLLGGHVAASLVRKANHSRLMWYNDDLLHMAVDIGNRLLPAFDTSTGI
PFPRVNLKYGNRGLKKQEVNTCTACAGTMILEFAALSRLTGNAVYEEKAAHALNYLWKQR
SRYSNLVGRVINVQSGEWIRRESGIGAGIDSYYEYLFKAYILLGETVYLHRFHTHYSAVK
RYMSGPHSAKFPFLFLDVNMHHPSERVRTFMDALFAFWPGLQVLSGDVKPAIALHEFLFQ
VYKRNKLLPEAFTPDLNVHWGEHLLRPEFIESTYLLHQATGDPYYLDVGVQMVNDLEQYA
RVPCGYAAIQDVRTMQHQDRFDSFVLAETFKYFYLLFSEPSDLPLSLSEYVLTTEAHLLP
LSFSPFHSMTVTEITTSVDSRKTTRAPPPTKTLPDKFGDRDHAAEAYLYRRGRCPDVNLE
HFYGSPNICPDNQNATATPAALKPYCDVLDGPGLHQHRLFWLQWLNGRPGRPYCQESSDK

QRWWHHIDLIRQPLRQLASVRKTTTQDGVYVDAIRMPLRAADFRPDDKAQLALLRRMGIE
LIVKNDGRLVFKHDQNMADSQALGVAGLLFVHDLVQLVSLHSEAEALSESTVQPRHVAIL
DPPSFGRLRFRALPAHFGHFPGESDTKSEDYISEPDSHSDAKLPIGLEVKQDRKTPSAWQ
PVVAPLRVAFPTDGCSEVEATGIAFPAASSHWNAPAADEQTGDQAHAKSPEAIASRGPMA
GAIGIVRRGGCLFVQKARNLAKAGAVAGIVMDHEPDSSAARAILFTMSGEDDPSKNDVDI
PFTLLFAAERDQLIAHMRQHWSETRQPTVAMLTKEFNATRVACRSDGWMQEAKSSGAAWS
PPIRSLATSRPVASSPFSIYGKVSSDNPRAGRSPDQIDSIEEIGGLIVRRLPSIPISCET
STNLNTCLSDSVRLTGTQWLVSLIVNQYEVLDYVPVPKDTEETNLPNKCVHKWIHDFERA
FSSLTLSESCRGLLLALFEALLRSASHSVDHKKIEVLNVPPHWISEMNRCLNYLKELSLF
KSVFGIYKHQQTCEQSQGITRTPVSLSISFIPVSYRIWTKAA*
>BN1106_s1832B000342
MLSPRWLPALFCIFVVCDGLKNLKVTRNIDLKSPVTRIELEITVDDGKADYEFLLNPSEY
SRLSWLSATPKGSQKELKVEKSSKPLTHVIHLENKPGQYEFVVLAVLTGQLDPKPKELLQ
GDNQYVKYTGDVYFYSHYSTEEQVTNILLPVGELLHHTSTPDPVTKSGNKLTYGPYKEKP
ALSHSPLNVHFENNRPFLKVNQLTRHIEISHWGNVAVEETLEIINAGAKLRGSFSRYDFD
LGKGRKSAVASFKTALPAAAKHIYYRDEIGNISTSTVSELLDAVEVRLQPRFPLFGGWKT
QYTLGYNVPAHEFLYRSVLHYFALIGSQFTLKMRFVDHIYDDQMIDHATIKIVLPELVTN
IEFLPPYSVDESPREVLKTYLDTTGRTVLVFTANNLVSEHIKDFTLRYQFNMILMLREPL
MLVAAFCAIFLCLIIYVRLDFSIFKDEKAELRMRIQAIVEEAQEFYQQRCDLYQSYEEIL
NRYKSSKNASQFSSERKRLEVVHKNLNHRLAQVQGKFGDLYTDGVEKIKEVISLDSRYRD
LIQECVQLAERLISGKINKQQYQTSTNDITSKKADLRSRMDALIENL*
>BN1106_s1723B000106
MTEDTRHYGSLVFKIALFANLISVLFNVINDCDETYNYWEPLHFIVTQGHGGGFQTWEYS
PSYGLRSYLYLWLVGWPAFLIALLGWPLWLGFLLVRLHLALWSVGALTYMSIVLDRVVPS
DASGKSFGLPLSIWFCGFYALSPGCFISSTTFVPSGPATTLCQLMLAFWLSGHMFWAVGC
VALTGILIWPFAAFLGVPLALHLIGSRRLLTLIIYACTWAVFLIPVTLSIDSYHFGRFVL
APWNIIRYNLFPSAQNVSSGSASQLYGVEPASFYVKNYVLNQNVVIGLTGCFLLFAFGQS
LYALFTRAFRGFKTTTTHSGPIPLYLTLICASPMILWNLIFFAQQHKEERFLFPGYPCIS
LGAAIFIYWFVQRIARLTRWLRVNQFFSTVVSLLVIIVFLLASTSRAVGLIRWYSAPIYL
IRHLPHPNQTTAKPLLCLGRDWHYFPSRFLLPGNGDRWSVGYLQSNFSGQLPGHFTRVVG
PQSTWIDSVRSDGSRFNADNREESDRFVAGGSAACDYILDRDSKPGPREKLYVADKKQWR
SIATRAILEPHSCSKIAVPDEPWHSWISQYPILCHLFRAFYIPYVSEVTNRPVTMHILER
LPRQAV*
>BN1106_s233B000265
MEKIFEELKFDNKPGGVWKQGFEITYNMSQWEREPLEVFLVPHSHQDPGWIFTIDEYFEK
KTRAGLDATLDILLRHPEARFIYAEMSFFSKWVSGLTPKSKSLVAQLLHNGQLEIVSGGW
VMPDEATASYYAIVDQVIEGHHWLWDNFG
>BN1106_s1713B000125
MVFPVFLLFVTLLASCTYATDHVPLLSGYITLDDRNLMKNFFESHIAETKDDRTKAYHVA
LGLSSLGFNSGDAGTCQFTGSVKTPEVAFHRASVAKLTGNAKCMVEIKELQALQKSFLNE
DASAEDVFYLVSAMKYGGLEVDKNKVLEIINKIKAKDSTPSTCFIHRMAYVFQTVVKLDL
SRAFLKPFFDAVNDVLDQADELNGNQLFFEKGIFTTTMASKGIADLLTSYGEMEGISEAK
LTKLINFLYIRRHSTNLRAAAHLAAALRAFSQGPLITPVSITLGAQPPVNFVESFATGGE
LYRASPKLQLRLFSLWGESLTANEVTVKGSGLYAIQGPNKVRVLTGPSERGLFSSPDDKL
EPNSRTTGSITVGQKMLLSFRLVDNDTDETPLTAHQAFVQFTHKETGQSITFVCHEIVAT
GAANAKAYQLKLDPESSSEDFDNLDGIYKVELFVGDSLFLKPIVWHMADLSLQFSGPRGS
DSARRIADAADVSRQKSPSAAGMKRANGLNPLIGTGPTKAKPGIEHVFRPPERRAPRPLA
WTFTALCAVPLLGLLIAETGIALTSTDLVMREAIIPDNEPDPYLRRLGAILVLYCVYWYS
LDMFTTLGYLCVLSLPTFLAGNSVLRAQLAARQALSGSNKK*
>BN1106_s244B000349
MFFQAAKLFKELTRKDAEKALTDEINRLVDTIPQNESTEKENFRTQMNGFQQLFQRYLHS
TSEAFDWKSMEPIPTECMKNYCDLARPTDRKTIQEQLNKLVVVKLNGGLGTTMGCTGPKS
LISVRNDLTFLDLNVQQIEVLNNDYGASIPLVLMNSFNTHADTEKVLRKYQQVNVEIVTF
MQSMYPRLNRESLLPLAKNAFVPRNNQSNPVESKPPKVDMSEWYPPGHGDFYRRFVESGL

AEKYLAAGKEWIFLANIDNLGATVDLNILNFLITSDPRPEFVMEVTDKTRADVKGGTLTK
YLGHLRLLELAQMRNNVAFQVPKDHVDEFASVRTFRIFNTNNLWISLPAMYEAVKAKKLQ
MEIIVNPKTLDSGLNILQLEQAAGAAIKSFNVAYGEPCYTLSD*
>BN1106_s254B000299
MFLQLNYSAVIIVEDDLDVAGDFFQYFAATLPMLMDNQNLFCVSAWNDNGRPGLVDPSRP
DLLYRTDFFAGLGWMLLRSFWLEINTNWPDIFWDEYVRKPYVRKNRTCLRPEVSRTITFG
RMGISRGQFFDTYLSTMRLNEKWQNFLQLDLTYLHEPGYTKRWLDSVYNKSMEISLDHFL
QGNLPNISTSSTRRLRVTYQSQADFDKIAKKLTLMRDIKSGVMRNGFVGVVPVKWKDRWI
YIAPPSTWAGYDLSWT*
>BN1106_s187B000154
MTIETILEAAKRAYQKCLRSSRLPKVQVKDFFFSHVRKSQISGRKNIYALLVSLILCGGI
YSFWKDSGKPLNSLSNSTTEQTDPPEIIFNELSDNRMSRVIKDAMVHSWKAYRMYAWGMD
VTNPIKLSGSEWMGAALTMIDSLDTLWIMGLTKEFDDARGWIAANLTFDKNNDHINLFET
TIRVLGGLLSAYHLSKDDLFLQKAVGPKSNFRADSQTKCLSFSSASLAHPFSLWFWVTLA
*
>BN1106_s1989B000317
MIMGHTVHRIPFPVLGVCFLIISFENAAFGRTQVVQLRALKEQKDLVNDHSQFYKNWSKQ
FRDLMKHHEQQYGIKQDTVRTIFGLCSTPKHNWPSALESSSFTIDRPEFCRVRPRVRYLI
AVHSHSKSRHRRDLIRSTWASLRRVGGERIATLFFLGRAENKQGQEDINQESAQYQDIIQ
RNFTEHYHNMTRKHLTVMEWVSKGYCESLEYLIKVDDDTFVDVFHLVRFLKTERLKTPNS
FYCSATSGARPIRPSKKTPSKWVITTTEFEKSVFPTYCEGLGYIIEAHLAPYLYWCSLFT
PPIWIDDVYVTGILAENLGFQLQEFIPGHAYSRVGPSKQNEHLLDSIFLTSYHSEFLPET
FRRLWQTAVSRSMDIF*
>BN1106_s2077B000122
MNGDICCDLPLEDMLEFHSRLGSGDRFVIMGTEATRQQSMKFGCIVENPDTHEVLHYVEK
PTTFVSTTINCGIYLFTPGIFKFIRIAFLEHQNQRTYELRAQCREIIHLEREICQPLAGS
GTLFVYHTTRFWSQIKFAGAVIYANRHVLSLFERTHPHRLARMTVPSSTGLQYLTGNTRL
PDLAMNGDCVLENVGPIIIGHVFIHPTASIDRTAVLGPNVSIGERAVIRGGVRLRDCIVL
RDAEIRAHACCLNSVIGWNTIIGEWARVEGTPNDPNPNKPFTKLDVLPVFNAKGQLNPSI
TVIGSNVEVPPEVIVLNCIVLPHKELSHSSKNQIIL*
>BN1106_s10435B000022
MIPSNARSSYISFILLRYRDRCIMASQMTYNIQTKHTKPFPDQKPGTSGLRKPTKTFMQP
LYTENFVQSVLTAGLHDLLEARQHVRLVLGGDGRYFVKESLLNIIIPICAANGVSEVLVG
QSGILSTPAASCVIRKYNLNGGIILTASHNPGGPDADFGIKYNCENGGPAPEKVTNKIFE
ISKGLTQYKTLGQPMELNLDTIGASSFQLDNGQVFRVSGSSQK*
>BN1106_s269B000233
VNVISSVDDYANYISTLFDFEAIRNLLSGQGGQAPFKLLVSGLHGVTGPYIKEIFCKRLG
MPASCALKAEPLVDFGRGHPDPNLTYAADLVQAVIDDKSVSFAAAFDGDGDRNMLIGQRG
FFISPCDSLAVIADNAMSIKYFQQTGLRGCARSMPTSRALDRVCMAKNIPYFEVPTGWKF
FGNLMDAKMCSLCGEESFGTGSDHIREKDGIWALLAWLSILARRQQIGKPVDVESIMREH
WGTYGRYFFTRYDYENCTTAQGEEIMSQLQALLREGVSNRVFDTTSGRQFKGQFCDDFSY
SDPVDRSQTTNQVYLQPHIELALKLCNVNKITGRTAPTVIT*
>BN1106_s3450B000051
MNAVLARVRWRDMCIILCGMITMKIILIACGGVNFKPTERITLHTGTAPVKSITSGESPQ
LERPRIYCILLTNKPNYELKAIHVQNSWARRCTEFSFASDKKHPHLHLLDFKLTKRDTYD
NLWAKMIDVYRRLYYLADEYDYFFKGDDDTFVIYENLEGLLQSFPSNRSIHLGYLMGDRV
NSLFYSGGGGYILSRRALKDIVERGLGFNEKSRKCNTIQGPEDTLLAACAQLVGVAQYDC
RDVNRTDIFSNRSPDTLFEHLRFTAPKLLKYSTHWDHFSENQVTTHYVEPAMNYVMEFLV
YHVGQLLKQNLLQIPTVYSSKANFICERTYLQRAN*
>BN1106_s309B000237
MSSGTCPSKFMTEITGSRVTVLLLGMLAGHVIHMGINLILQPKYTALPGAQAIIKPKNIR
VLCYINTFPANYELKVIHVYYTWARRCTKIWFTSTKPHRDLPIMILNLTVPETRMHLWSK
MRRILRQLYMERNNYDYFFKADDDTYAVMENLRVALAENSPEKSFMTGFRWNTLCPGGYF
SGGSGYVLSRQALTQIVEQAIDKHPNCPTYDEDKEDVKLCKY*

>BN1106_s452B000151
MYPPSEESIKRIEKMCSKSISFEKVDLVDYEAVEKVFQKYDIEYVIHFAALKSVNESVTK
PLLYYDNNVTGLLNLLRILEASCFHYSCDRWKLMKDESKNAIFTGDYRNQLPFVTSLFDE
KRKHPDEKVMDSHKKKNLVFSSSCTVYGDPKFLPVTEDHPIGDCINPYGASKFFAEIILK
ASENFIGHSYKM*
>BN1106_s2686B000193
MAIRKLWGDDRCWGGRKVRHVFLLGLLNNQTAPRLPQVDREIEIFGDIIQQGFIDHYYNN
TYKMLFGIQWAVAFCPEAKWLMFVDDDFFVNPRLVLSFIDSLDPRLQTKLVVGDLAVKAA
VLREKSKWSVNKTLFSHRSYPNFVQAGAFFMGAPMAVDLYVGSRFTEFFPFDDVFIGLVL
NKLLVAPAHMRGLLMYQPNFRKRLILNGSLALHGIRSAERQKWLWHIARLRDMCRVSK*
>BN1106_s273B000155
MVLADTMAINEEQTMHQDFAIKQKCKSRCRSCSEMEYIPIVLMKALILVGGYGTRLRPLT
LTHPKPIVEFCNKPMLLHQIEALVEVGVKQIILAVSRCADRCEILESELSKHEKRLGARI
TFSYETEAMGTAGPIALAREWLLCDDSPFFVLNSDIICEFPFRDLIKFHLGHGKEGSILV
TQVEEPSKYGVVVYEQETGRVDRFVEKPIEFVGNRINAGIYLLNCSIVDKIPLRPTSIEK
EIFPQMTKAKELYCFTLQGFWMDVGQPKDFLTGTSLFLNHLSRTSGDKILAKGPNIRGHV
MVHPTATVSPSCLLGPNVVVGPDCVIEDGVRIRNSTLLQASVVKAHSWVSNCIIGWRCCV
GQWVRIENVSVLGEDVVVSDELFVNGARVLPHKSILQSVVEPQIIM*
>BN1106_s3804B000160
MLSIQKLLKSSSKSLWLRNLELASVSLVIGFVGQIYSDGSLIRQRGFFYAFDWLVWLTIF
LHSFGGLIVALVVKYANNMLKGFACSVSIVLSCFYSLIFLGMQLNISFLVGTICVLTSVL
LYSMYPPPKRQTAQQ*
>BN1106_s2815B000111
MMRPILVAVFFAITIGTSFQFGYHTGLTNQPLELLINFIGNVTVERHGSTDGVHITLLGS
LCVVGFTIGGLVGGLVGGYLANRLGRRNSIFVLAAPCLLGCVLMTTSKVARAFEMIIVGR
VLVGFACGAYTAIGPAYLSEVAPPEIRGAAGVLNQLMVVFAIVLVQVLGLKEALCTEELW
PYLFGLNAIPCVISIACLLFCPESPRYLYLTRNNEEAARKALLRLGRKPDEIKAELNEMH
EETQNVTQKIALLSFFRVRHLRWGLLIALVCQMGQQLCGINGVNSRPLLYYSAELFKSTG
LSSQDANYATIGIGCVFFVVTLISVFIIDRVGRRVLLIGGLLTIFVCLVVYTICLVIRTY
AGVNWPAYVAIASTYIFIVGFGIGPGKSVCCVKCI*
>BN1106_s422B000584
MFLNSIRSRKVFRLIWSLTIVATIVVLYSFETAYLSKSSEFFALISTVKNNLTKRCKWPP
DSIEQLFDPTTGRYDLTLRPCVHPSVGVVDPCTTDDYKRLLSFYHGPPLTNKSCLRQMVE
MDEHVRMNREEPNYSLYPEMGNVLELVAAIREGKTVKTMQFEGLRVRNPTKVFKDAVATR
ASLLNESDHFRDMLIGDFIDSYYNLTLKQVMTFRWVSAFCRYTSPVYFFIDNDYSLVPSN
VIKMIKRTPDKLKLRLNGGTFGPLRAVLRPTNPQNARRWDMSVNEIPWTNYPQYSSGAAY
VVGASLVTDAAIAMAYTRFLRFDDAYLGFVWNKLHAPVMIIPGMKHTVIHSPLTEDAISI
PFREADQLVDWSTGTIRKKTPTDTKLKKKKE*
>BN1106_s3311B000068
MFGIVKWIVTHICRRIPHICIRPLAFISSGILVGLLISSRITKHIEANFPVDEIAPKAVS
FLLEPGSVCSSKYEIDWSELYELQTRVKVLCYVNTIPMTYKTKARHVMNTWARHCTKHLF
ISSEESTELPVINMNMSHPESRAHLWSKMQKAMRYLYQFRDQYDFFYKVDDDTFATVENL
QYALKDLNPDEPIISGFPFTHVIDKGHLSGGAGYVLSRATLKLLVEKALGKHPECPTYDE
DLEDVKLSLCAYAIGARYVPILDRHTTIPYVLDYPYVNEQRFQWMDLLTFFEYEQIKKRP
TSIPQFDPQNPFVRDEHGSTLLVAANRCSVCNGLHALEERQRFLSRDIPGHWAFFKEIKA
CFSCFTCGHLSSSCLVSKPCSTSGCQKRHYGLQLVAQKQCTDRSTHVTTQGLQLDKMGTD
DARKPGQWTSNCAATLRPECRASLGVLPVLVDRPGGRLCGDLRSAGQRVRCDFNTRRPYG
AGGPEDIPDLTFDFDRKWDYHLEFGNNGAATELIVRE*
>BN1106_s3542B000068
MSVYRPLFWLLEAPKVKIKKPAFVAWPSAMLLFTFVMVSYFLITGGVIYDMIIGPPSMGS
DTDERGNRRPVAIMTWRMNGQYILEGLAASFMFVVGSLGFILLDKMNETRMTKINRILLM
ALGIGCILLSFITLRIFMRTKLPSYMT*
>BN1106_s3389B000155
MLQTSVHVIVLGDVTRSPRILSQACYLANEGYTVTVSGYDASLSVAAKTKNINALDLVNI

PDLKNPVILVQSPPAVPTFLVLWIFTRIAGKKLIIDWHNYGFTLLELTTTRGGFLPRVYQ
LLELSFASRFLNSNVEHLCVSRALQKNLLRWNINAIVYYDRAPDDFASTPVDAAHELFTR
NNFAAHNNHTVVLLLWLLLKQYFFVVENTPVSDFLNAVVRLKTEYPALSDQVGSTRRTRF
TEVITLPADCGGQITQWRTDRPALVLSSCSWTPDDDFTMMIEALDKYNERAGKSESELPH
VVFAVTGRGPLKSYYEQLIREKCWTHVEVIMPWLTPEDYPVFLGCADLGISLHRSSSGLD
LPMKVVDLMGVEVPVLALNYETLHELLPEHKFGDHFLTATELCNQLCDLLKPNQPKSVRK
PLVVDRFDAIGSTKLRMYRDALSKYNRNTPRGLKYWKKVGAPPIYRALKSSL*
>BN1106_s4397B000042
MQSYVDKLHNALWKISEMVLRIVLPCACILLVGYLLFSYTPPKEQGKPIKQWTEYRLIEN
STYQIQTGTDDICPTLISKTTNELFAELLTKTVRALPMNFSARNVHQCNLFTQIYGDQLE
VSQEEMEFPLAFSFNIHKEFYQFARLFRAVYRHHNSYCIHVDAKADETFRRQVGDLATCF
GPNVHVIPLRQSISIHWADFRTVEAWIQCAKFFLEQSPIQWRYMLNGSGQEFPLLTNWEL
VKALKAINGSNIVESDYSPRFRSRIPKKPLSFKLTWVKGSIYTALRKEMVRFALTNNYAK
EILAALRSESKQKLCQDEMFFSTINYNPYFKAPGGCLVAKNPNDSDPRSTFVARYVDWYP
KPCLSKLSQREVCIMGVRDIPKLTKRYEFFVNKFLPDFEPVAYDCLEWWLFRKIRDERDF
GRTATSFNASFYSDLYCSSNHL*
>BN1106_s3686B000164
MLCKWAHSFEMIIIGRLIVGLACGAFTAVGPAYLYEVSPHTVRGAAGSLNQLVCVFSLLL
SQLLGLKQAMKTEQLWPLLLGIDYCLSHQHRFRRTERGAVRSDCLVIIYHADAHEQASSF
MSQFITLTCSSFACCATYQLLYYSGTLFTQNGLTTEQATYATIGLGLALFFSSLVSTLVM
DRLGRRVLMIGGLLISFFSLIVFTVCLIIHDSIGAQWPVYIAVAATYVFVIGFGIGPG*
>BN1106_s3082B000278
MNIIKRNLGKVTEINEACHVFQTTGANRSKTDRRKPKIRWKSGPKPIGFGAVFGGFLNGF
RAVCASRLKYVTSFLTSNLYAVTMLPSLFTHFRTNNETNLEFVVSADICRCDFGIRYSRA
QSKQTSCHVCTKSRYPFKHGYVRRVGGFKTPKLLHLIAFNGVKSAEGLFFMSLKQESVPA
FAHISPSASVYKLQPSDYMDIRRTGFSANAISDWVHSQTKIRIRFIRPPSYSAVILLSLF
MFIGAVALWSQKINFDGLYSSSLWCMLALTVIFGAISGQVYNQIRGPPLLHATPKGEIKA
FIYPGSDYQFVAETGIVMVLYMLCTAGIVLIHKVTETADAGKKKGQTCLSKVLLMERSLP
AA*
>BN1106_s4280B000148
MEELREKAIDEVMTKEAWNAYAKYAWGANELKPLSKKPHQASVLGGVPLGATIIDGMDTL
YIMGLHEEFKNARDFVAEKLNFDQVFLDQAVNLADRLLPAFNSTTGIPYSLINLHDGFTK
QFSWSLDRCSILSECGTMHMEFTYLSELTGDAKYAKVVNTIRESVSQIPRPNGLFYNYLN
PRTGSWCGQNAGLSALGDSFYEYLLKEWIRTDGADIQGRQLYDIGLQALFNNGVFKKSAA
DNLFLGSYHHGTVNSVMDHLACFAAGQQVDVEQGTECDKYELILNDDKTGLLPSFQRGKS
SSSSSSSSYNDKQYGKIHCGMLALGSSGPNDVWFDRGIRITNTCRLSYENTVTHLGPESF
EFGKNIEAVPIRKAHKTHLLRPETVESYFYLWRFTKNPIYREWAWDVVQALISYTNTSTG
YSGLIDVNRLQKNWDDVQQSFFIAETLKYLYLIFSEDTLLPLDRWVFNSEAHPFPVHNRV
ILGDTNWPKLHPLTKQPKPVMPPSQSSISSHRPPPLPQSV*
>BN1106_s2582B000094
MIRLSCSFKTYDWGKLGHESEVFNLLSGCTEKSDLPNDIPFAELWMGTHLSGPSHIYGDE
NQSLATFISFNPEVLGRSGKMFGRTLPFLFKVLSVRKALSIQAHPNKSHAQELHRNRPDI
YKDANHKPELAIALTPFEALLAFRPVSEIACFVQGIPELLEVIGIASATDLSSVSDDTEC
VAIKAAYERLMRADVELISDRVNRLHQRLKSGSKLDLTLPESSRVNLDVLREVFIRLADE
YPGDCGCFSLFFLNYIRLEPGQAVFLEANLPHAYISGDCVECMACSDNVVRAGLTPKFKD
VDCLLSMLQYEPRTNNRIIFPPKECSIRIPDSKMGLTRDLMPTVFTYAPPVEEFAVDRIC
IPAKCEAFHLFASPSASILIIISGSGRFQVFLPKDRDNENPKDPRSDSLTEEKGSSYGSE
KGEIVYAGSVNQMIGFHRGMVFFVHANVSFSLHPATPNNDSILAFRAYANVDMISQSEDT
IYDKTSSVVLGQARQM*
>BN1106_s3059B000208
MRLVSSKLFFYLLCCNVVTCTNSEEMAVLRNRVKRMFYHAYNGYMTHAYPLDELRPLTCD
GHDTWGSYALTLVDALDTLVILENHTEFRRASRMLLDHLDINRNVNISVFETNIRVVGGL
LSAHLFSRRAGFEVESTWPCSGPLLRYAELFASKLLPAFDTPTGMPYGTVNLASNGVPLN
ETPVTCVAGVGTMILEFGTLSRLTGDPRFEAAAMRAIRALWRYRSSIGLLGNHIDVLTGR

WTAVEFTIGGGVDSFFEYLVKGSILLRLPELDAMFREYKKAIDLHAKHGDWHFRINKDSA
HVTRPLFQSLDAFWPGTLSLFGHLDEAIQHLSAYHEIWKQYGFIPEAYNLIERRSVPKQS
SYLLRPAVQRMCDPEVRTRFEQKLAHELGSAMNSIVDEQRAHIEQIRCPSKTLDLARVAE
YNGAKASTQQLLFPLAFLSTEFIESVYYLYRATKDPVYISMAIDVLTSIEQTTKTSCGHA
TVANVVTHQLENRMESFFLAETIKYLYLIFDEDNFVNHIPGDHPAPVSHLSSAGVECTLE
NGGYVFNTEAHPIDPGALHCCSPKLMSPSSSSATATEAILTAQHHPKQNTVSAEDLERLS
REEAQYQPTEELLNLVDSVFAEHLNIDPDMTDPTSCISFEPTPTRKPCANLSSINQALVE
PWLDLRQFVLQLTNDTLMKNHSSGTVGSLDEDSVITDFNAFEPPLLTCPYPPFHQRYTYS
GQMVITEN*
>BN1106_s3158B000070
MSSDPLNHVILVTGGSGLVGQGIRLALDNPEYNLRRENEHWVFASSKDVNLTDAKATQQY
FEQIRPTHVIHLAAKVGGLFANMSGNLEFFRQNVHINDNVLASSFSVGVRKLISCLSTCI
FPDRTTYPIDETMIHNGPPHDSNFGYSYAKRMIDVLNRAYAEKYEVVYTSVIPTNVFGPF
DNFNIEQGHVLPGLMHKAYLSKQRNQPLVVWGSGAPLRQFIYSIDLGRLIVWALREYNDI
SPIILSVPEDQEISIRQAAELVALAMGCTQLTVSFLAHNCDCFLIVLLFSLLSGFHFDTS
KADGQFRKTANAAKLRSLYPNFIFTPFEKGEHWELLFLTVCLKFD*
>BN1106_s4527B000076
MALFARTVARLASVVAQYAQTESGQARLQTLEHARLLSAEYSRWADLLFDPDILDQLHWS
DEAGRYADYGLHTDAVHLQMPARPTEPRSSPENEEEPKPIRITDKPPTLKLVTSSAGYLS
LFPLLLRILPPNSPRLDRILSELESDDIWTEHGLRSLSRSSPLYMKSNTKDDPPYWRGAI
WINMNYLAVRSLRYYSANARTPTDVASRAQKLVVRLSRNLARTVLGELERTGYLWEQYDD
NTGRGQRVHPFSGWTSLVALTISSDAY*
>BN1106_s429B000289
MLPYVRCLSGRYSPRVYVIANTDKISEDRLHAVEQLKEGEYTVVRIPRAREVKQSYVTSI
FTTARSTISSISLVFHTRPRLILCNGPGTCIPVCFAAVLARVLLFRQTLIVFVESVCRTR
TLSLTGKILYYSRCADVIVQWPQLHAAYPETVYLGLLS*
>BN1106_s4527B000077
MRVHVSLAMPKSKVIDVRPKKRKTLHKLEQSSTVVSNENPKKIVTHNGNHLVDKFRFSKA
KSRGHGDKHFSSELHFGKSQWLIYFGIVMCAVLAVVLVSTGVHRWFSWRQAQSIKTPLNL
PLVIAEDTSPADLFWGTYRPGFYFGLKHRSPQPLIFGLIWTVQDIRNPRFRHVCDSHDGV
LRQTDPPQPLSVIVYLYYSSAAGSPAFAPVVESEKVTGLKGFTSALGEHQIFIHPSKENI
QVSSLIALVPSEEQIMETMLNGVGLRRDTNMLVLTGRPKGYDMNQMPNIWFHEVTVTPPI
MTNKVDAGQIVMEVEYTQIGRKGGAFVREEFTRRLNELSAEFHKRFTERFPVDLSQFTER
QANLSKISVSNLLGGLGYFYGSSLVQSPRPGSEPVSNWPAGLFTATPSRSMFPRGFLWDE
GFHGLILARWDPILAMETVGHWLDLMNMDGWIPREVILGSEARSRVPPKFVVQQDNIANP
PSLALVIDALMDEVPKFSSAEVTRFRQWSLAALPRLHSWYQWLNRTQAGPVPFSYRWRGR
NSQELHQLNPLTLSS
>BN1106_s2625B000068
MRPARRKYAKSERKQDATIIVWRKKRLLITGMLWMTTSLLMAILIRFTFPDCDKMFFMSI
NTKTEEISRQETIQSERLLEASNKLDNSWLNSISRTQAQNLNKYNGLTLHHMGQKQHSND
HIHLKDDYRERIHPIIPPIRSNQDSAGPGEHGLAYEVDRMFLLPVEQEEYDWGWKNNAFN
QFASDRISVRRHLPDYREGTCLTNLYPKDLPSTSVVICFFNECWSTLIRSVHSVLDRSPS
HLIKEIILVDDFSDLKHLKKPLDDYVSALGKVRVLHLPKREGLIRARMIGMNASSSDVVT
FLDSHIECTEGWLEPLLARVHEDKTNVVSPVIDRIVDATFEYTVLHANEVQVGGFDWDLT
FDWHVPPTKDKLRPGAPYSPIRCVHILSRYLLVSHII*
>BN1106_s4461B000061
MFYTPAIRIFSLVLIFLACEFLYPFTPFINLLCAAAGVKESRDSLWDKVKFGVQMVIDNK
TKDYDYFLKADDDTYMIMENVRVMLDGLNPEQPLIVGRRFKKFIREGYASGGGGYIISRA
GLKLIAEGMKTNPKCMVHNHSWTEDVFMGICAANVGVKFIDSLDQMGRERFHPFIFSMMR
DEKIPSKGDWLAEYNYHPILRGVGCCSDYSVSFHYLNPSDMITYDYLLYQLRPYGIHHDY
KDVVKLLRNYSAFT*
>BN1106_s373B000291
MTDQRQTSFQQCCFSAKFVFPNFKNIFIHETRFSVLFMLEICDNQSYLGCLAFGYSSAAA
FQIFLNVTWSSVFASCLNVGGLVGSLSSGYFLSKFGRRWTLIAGCLPGIIGWIWLRMSSN

GVYERYSPTVLFICGRVMTGVSAGMTIPASASYLAEIAPPDWYNVFGTLTQLGIVCGIAL
AYLLGALLPWENVALIDSILMVVLLLCVLVLPESPKWLAKAGHLQQANAAQIWLHNSHSR
TLSTSDMKTVEDGDSVEPETQHTFWDYIFPCRAIPPSQYSRLRVTALLMAFQQLTGINAI
LYFAESVCLFAGLSWASTCALTLGIFQIIFTLISAPVVHRVSRRKVLMWTAVIMSVAHVL
HGCIFLVLDDRNLKFAAVGERQVTSVESHVWKG*
>BN1106_s2843B000179
MVANRVLPLSFCYAVDILMGIAGTGSLSLPMFSALRRLSNLFIMVGEKVILGVSRPFSVY
LSVVVMVLGAIVAVIGDLTFDLLGYAYVFTNNFSTAAKALLTKSLLRDQGFSSLELLYYN
SGLMVPALLIIVVLQTDLYQLNYLLNLSALWMESEM*
>BN1106_s2903B000171
MSLLKQFFVAFDSCISNSVVSHRDFVFNHHSLFCFMPFSCRLRRLMQRRFGRVGTILIVA
LLLLITLDIWGYWERPQFAESLLNGNITWLLTDCVSEAALVECSPDNKRLSHSLPSSAVS
VSRQADQFNTEQFVTNQKLFGRLPSPIEPSLFSSPDAVFLVQVHNRFENLRALIESLSRV
RGIEKALLIFSTDFFSGEFVSLIHAISFSRATYIYFPHNNQVFPDLFPGRDPRDCEANVS
VQQAQRVGCLNANWYDRFKHYRDSRYSQVKHHWLWKRIANSHCCIIVTYIHRRVSTSFLL
ISYYEQLVTGTFSVFSLFAAIFPQLAFMFERLHAVRHYDGYVIFLEEDHYVVEDILHMKK
LVETIWKPNEAKGMIAFGSYATQQNYKDPQVAFGPWISSRDNMGMGISRSMWNRIKPCLA
SFCTFDDYNWDWTLQHIGANCMLPRLEAMQLLKQTRVYHLGQCDGLHHTAANCSVRLLAQ
KITQTLQGPDAAYLFPSKLKITELHKSGLRGRPNGGWSDLRDRALCMSMATGVWQPDIIE
YAPHLTQHSAL*
>BN1106_s384B000400
MKSDDFLRLSLPVLLSLTLRSSVLLHPHSGQGKPPMYGDYEAQRHWMEITTNLPPSEWYF
NSTKNDLNYWGLDYPPLTAYHSWVLGKISHEINPAWTSLTSSRGVETKDHKLFMRYTVLL
ADLLVFIPAVLYFFYVALPTVAPSSEVPPFYACCLTFMYPGLILIDHGHFQYNCVSLGLF
ILALGLILRNRDISGTVFFCLALSYKQMELYHSLPLFFYLLGKCVHSPASLGMIRLLKLS
AAVLSTLALVFTPFLTNTTTLYQVFHRLFPVARGLYEDKVANFWCATSPLLKWPVLFAQM
DLVKLCIASVALLSFPGCLSLLVRPSRRRLLYTLSSTSMAFFLFSYQVHEKSILLVAIPA
LCTLPICPLPSFLFALSSTLSMWPLFLKDQLVVPCLCTTLIFVSMGSIVLMRSPSSTEPK
SSPASVYSLTLLFMILSSYGVIFFSQALWYPPAAYPDLFALLISALSCFQFVVFLLFWNY
KSFAMNG
>BN1106_s2625B000067
MGFALRRSTPTIAGGLFAIHRDFFAKLGYYDPGMEVWGGENLEISFKASLCMAIICWTGY
FTDVPDFRQTWMCGGTLEIVVCSHIGHVFRTRNPYMADRAGEHALKRNMVRLAEVWMDDF
RNFFYDRFYFRLTHDNLLTFPFPLEQGDYGNVSDRKAIRERNKCHSFSWYLDNIYPELFV
PSKAQASGDKVDITLFKAYFHFIRTHNLLPDCRCTFSSLGHLHIQIENFAAPVCVDASSD
PKLTELHVIRPYGCHRLGGHQLWYLSQLNEIRRDKMCWTVGDDNEMVGMVNCHGLRDTQE
FTYTQENLIKNNGLCLELTENYDRIILAQCTGILRQRWKFSREPVSPPTSSTHPIPAALF
GYYGMDSAEKL*
>BN1106_s3727B000148
MKVPIRLDFGSQVSLIQLGVLLLSAAMAFSIRLFAVIRYESIIHEFDPYFNYRTTRFMTE
NGFYEFHDWFDDMAWYPLGRIIGGTIYPGLMVTSSLLFYLLDLLHVTVDIRNICVFLAPL
FSSFTTVVTFLLTKEVWDTKAGLLASALIAVVPGYISRSVAGSYDNEGIAIFCMLFTFYL
WVKSVKTGSVLWGSICSLGYFYMVSSWGGYVFLINVIPIHVLILMVTGRFSHRLYVAYST
VYTIGTILSMQISFVGFLPVISSEHMGALGVFGLIQLYAFYEYMRTKLSAAQFHQLLRIL
VAIVGAALCVGGTALALSGKIAPWTGRFYSLLDPTYAKNHIPIIASVSEHQPTAWSGYYF
DLQMLAVMFPVGLYYCFKKLTDSSIFVITYGILSLYFSDARIMSWWDYGYQITGIANRTV
LVDNNTWNNTHIGRVGQAMASSEEEAYEIMQELDVDYVLVIFGGLLGYSSDG*
>BN1106_s3991B000079
FGGTVSVKELVKFVDEGGNLIVTGSRDIGEAIREIGVECGVEYDEMGTAVIDHHNYDITD
DGTHTKVVVAAENLIKNSIIIGNASKSGLLYRGVGSSVSGNKQLMDNLVPWVFGERGQLR
VAGVEHHLAKQHLIPSQYTIMDEVYYSIIIETKSDSGDWVPFKTDDVQLEFVRIDPFIRR
TLEFKGRNTEKLFSFSETLVVPWLNHQFTVPVRPFTHTQYERFIVAAYPYYFSAISMLVG
VVLFTFIFLYYRDEKEKSD*
>BN1106_s4335B000092

MPTELSTYASAVAFGQGHLFAFWDLLTAVEQGSLLSDVESIDFDKIAQIFQGSQKQTKNN
IEERLLPPEDDICGCQCELRKSNPALLERYYQTGRFTTADCSIFKKALSAVHEGKVAVLL
LAGGQGTRLGVNYPKGLYKPGLPSGRSLYQIQAEQIRRVKQLAAQRFGSLPSIPWYIMTS
EHTQDTTIAYFQSHNYFGHDPKDIIFFEQFNLPALDFDGKILMSSKSKICFSPDGNGGLY
KALRERHILGDMKSRGVVYIQTYCVDNILVKLPDLHFIGFCMDRNAECGAQVVQKVNPKE
PIGVLGMVNGRYQVSKTKILFCPYDTIKMLHSFSNKEVTYIMKEVVEYSEISLETAALRR
SNGVPDQISCSQSDGNDPASSNERLVYSHGNICVHFFTRQFLERVTHPDILNLMKHHVAR
KRVAHLDLATGQQVTPSEPNGVKFEKFIFDVFPLAERFAIWEVPRKERFSPLKNGPDAKM
DCPQTSRADYLSYHAMLARSAGAKLAGENSTFHSVGETNESETRALLEISPLVTYEGENL
DCLAGVELHGVNVLELDDRTGKPSLRLVDQKRGEL*
>BN1106_s3727B000147
MSLLLSNLFNDHELCGGVYLFVGFSVHRRFAHINKFIWMVRIAGSTEKGRHIREEDYYTP
HGEMRVDKSATPTMQNCLMYKLSYYRFWELQTDRSKPFGFDRVRGQVIGNRGYSLTGLEE
AFTSENWLVRIYRVKPPANRGTL*
>BN1106_s3078B000149
MNTHTGPFHLLCNYLLVIVSAISFKLLFLFGYRSTDFEVHRNWKALTYSLPISQWYFEST
SEWTLDYPPLFAWFERALAQIGSYIDPKLCVISKEPYVSPQTVVYLRSTVILSETLLFFA
LWRLCKALSNSSSFTMKRKFISMAILLAFNYGLIIVDRILCIETGCVIILLEFGLFTLVY
CKQRSLSPTDEEFFLHNSYYQFAPTVPSKQASMTGGLVGEIQHSILPSVRPLHTAMLTFI
GMCPTLIQCARARNGLEKEIVSRNYVQFLTGLTGAAWSSFLFGWHVHEKAVLMILLPLNL
LATVALKYRFIAFYVTTLGHYSLIPLIHTAADHWIACVENFPPFSFVVTCRQVFEASPKT
EGLKLLDQFSPSSVPESHGRCVRATIEQ*
>BN1106_s4192B000062
MACFPSDQFMMLYLRIAFSAAADTEDRGHLWTKTKNGLHHAMSHYSDYDFFLKADDDTYT
IVENLRFLLKDRDPEIPIIMGRRWRDFRGDGFLISVSIRLTVSLAGTRLHLPQECKDYSR
GINGRRSSDSSKTELNPLTFVARCGQLHDKAIMFEPTEKLLVHNQAQPHVKQGYLSGGGG
YVMSRAALKLIIAGLKMDPSCAGTEAGGAEDVRLGRSGT*
>BN1106_s3970B000071
MRYTGPGLRVVPGPLLTMTLSTGMSCCMAALLSIVTVAVMNLCGDLLSSSKGLTIFGGFL
GSVLFLLLITMVNNAECLVFGEGFQSGLFPEGK*
>D915_15676
MRSHRDGRAGFFAINDSVGLPNTNPESADVERFFPSNHRVNLTKQRGKYDLALQVQNELV
PNRRLNSCEVIATFSGHVPWISPEEYRYPLAFAISVFENPEQFAHFLRLIYRPQNVYCIH
IDRKTERSTTEQFENIAHCFGPNVFLIPVDQRVDVAWGYFSVLQSTLLCAEHLLNQTVVP
NWKYMLNMNNKEIPLRTNWEMVSALKALNGSNLVESVPCPDLEQRKPKHKYTFKYGFKIW
MHIRSVFPSM*
>D915_09300
MPGVRIDGFDVAVLVIYFVGVLIAGFASMFASKRGTVSGFFLAGRFMTWIPVMSLQKLTS
AFKGRSFIVR*
>D915_14056
MLLSGLLGFAIGYVSALQIQVTSPLTHNVSGTAKAAAQTVLAVAIFQEVKSSSWWLSNLI
VLLGSAAYAYVRQKESAARQNCRSSVDGQSDDVNSSVRTLNKLPTPMTKLSVI*
>BN1106_s741B000214
MSSLFFVYNNQANSGRGNERFTEPIRKFSWLSPEDLDQCAIDTALSTMNFTLNLTIPDWM
ELLSEYVPIKRHESGWTVYRLPNNQYYLDNQMIIKSNKTANHSKIATFGQNLLLARVNLS
LAIWNPMLCKPQVSTAIIIPYRNREEHLRALLHTLPAFLIHQNVRFTIFIIEQMSNTRFN
RALLFNVGFIESRRIGNFDCYIFHDVDLLPADLRVPYSCGSQPVHLAASMDKFGYRLPYR
NFVGGVLAMSRKQFTAIGGFSNYYFGWGGEDDDMFNRIKQHSLSVVRPKMTRYRMIRHGR
DKLNERNKENLQVLTSTQRKALDHDGYLQADYQVLTAGPRYSGLVYWIEVDVHERKIIAQ
RDAILKRT*
>BN1106_s9586B000050
MIAFYVPLRRIHKPKNELADYQVLTDTGTLWLPKSLTQTEVICPILISPKLNEQFADTLT
KITRTDQTNINPNQIDDCPQFKQIHGDQPSVSDEELQFPLAFSFNVHKQFTQFARLFRAV
FRHHNAYCIHVDYKADREFRKQVMRLVNCFGPNVYVIPRERSISVKWGDLSTLEAWIRCA

EFLLNQSSFTWFNGSFYTALRKEMIQFALRNKYATEILTALRREDHRPKFQHELFFSTLN
YNSQFNAPGACAEVHRPTQPDPLSTFVARYAVWLPQPCLSERSQRNVCIMGVRNIPTLTN
RYEFFVNKFIYDFQPLAYDCLEWWLFRKIQYERNSGHTASSFDPSFYANLYCSKNHL*
>D915_04597
MLITFPQSPMGFALRRSTPTIAGGLFAIHRDFFAKLGYYDPGMEVWGGENLEISFKASHF
LLKIYTSVAELPPPTHSLPQPKCLCMAIICWTGYFTDVPDFRQTWMCGGTLEIVVCSHIG
HVFRTRNPYMADRAGEHALKRNMVRLAEVWMDDFRNFFYDRFYFRLVRSFCPFIQQLLMS
RHTQFYVYDRSIRLCFVLFCP*
>D915_13377
MRVILQRIYQFRNQYSYFYKADDDTFSIIENLKHELANHNPDDPFMTGHRWHLRIPGGYF
SGGAGYVLSREALKRIVEKAIFKHPKCPDTDESMEDVKMST*
>D915_01292
MQTRHADLGDASDRVQSLDDVYYFGGQQASPFEAVIADQPSGIQPGDLVHMAGNHWNGYA
KVTPVKLPDAVLAPAYKFQPRVLAVNMGNHSVL*
>D915_15656
ISSDPANPLLMSVLRASRTAYSYSPSKSVTDYPNSVGTNTHLIVALQARNNARVLFLGSL
DFLSNEFFRSPVKNAVSGV
>D915_11722
MFASKRGTVTGFFLAGRFMSWLPVSNSTVICSALFQIGASLFASNIGSEHFIGLAGSGAA
SGIGVAAFELNVSVLPTILRPIHSCLFYEEDYSRCAIELGFAVVTASRLGIFAGLYCQWS
IYASGLHEPSLWGKSNSDIFSRSLTATLHFHKNIDLLIG*
>D915_14688
MLPGTLILKCYVLLFSLHFCQANDVVWAVNCGGSGHVDSNGIEYMADPLLTGTSSDYGRS
FTINRVPPEDQILYQTERYHTGDFSYNVPVPEDGEYVLTLKFSEVWFTDPDQKTNRDNPK
VNALILTRGSLSDVPQLPPLEETSKPPQRITPVPDLDTDDELRPRRPAGTPRAPDPYAST
EYSYLLLPILVSIAAFLPILFCLCKI*
>BN1106_s5562B000030
MPTATHVHRFASPRKVRQWCLVLLVFVVIVLLIKTWPKSIITSIRYRDSHELFKHRTDQL
LRKEFVGLIRDDIPAVEDVGEAMLNEWTALENQFCAQIFSNRSYSFATDACRYSCEHKDY
GTGTSNEYGRRNLVVIPFRDRHEHLKQLVPRLEHILQKQKICYLIVVSEQIGTEPFNKGM
LMNAAFVEALRLFPFHCITLHDVDLLALSDDTPYGCPTFPQHTSVHIDKFRNRLPYIELV
GGILSLPIKVFLRVNGFSNLYWGWGAEDDDMYERLPYIELVGGILSLPIKVFLRVNGFSN
LYWGWGAEDDDMYERSQILTLGEARYRLDGLNSLNYTLVDQYVKFYHSSDENATNTKGRS
NQSTDQHWLVHLKIDVGRAPSWLRIASVQVKR*
>BN1106_s639B000755
MTSPTGDQNQKVALITGITGQDGAYLAEFLLSKNYEIQVHGIIRRSSSFNTGRVNHIYEN
DRNAQTRRFFLHYGDMTDSSSLIKVISEVSPDEIYNLAAQSHVKVSFSLSEYTGEVVALG
TLRLLDAIRTCKLERKVKFYQASSSELFGKTESTPQTETTPFHPRSPYAVAKLYAYWIVV
NYRESYEMFACNGILFNHESPRRGETFVTRKITRAAVRIRRGLQDVLELGNLNAERDWGF
AGDYVRAMWLMLQQDKPEDYVIATGEKHSVREFTNLAFAHVGIQLRWEGTGLNEVGIDSS
TGIVRVRVAERYFRPAEVDQLLGDSSKARARLNWFPTCSFDQLVKMMIDSDMDLLESGKD
VM*
>D915_10315
MKAVILIGGYGTRLRPLTLSIPKPIVEFCNRPMLLHQVEALMQVTWYFMVAVKFQIDVDE
IILAINRQAAALEPFIKESCKSVIRDKDVKITFSYEDEALDTAGPLAQAAEYLDSDNESF
LVLNSDIICRYPFKKMIEFHLSHGHEGTMVVTKVEEPSKYGAVVHNDQTGLVKHFVEKPS
EYIANRVNAGLYMFKPSILSRIQAKPTSIETSIFPAMVSEGELYCMELEGELSLF*
>D915_08494
MLFRNILTLGKLLICLNVSLFLCPIQIVQSRPLKSPTFDQGQICPNCECWMLHQLNGLVH
PHPRTISDLQSWLPKVSIVIVFHNEEPANLLATVFSIWNTTSTNLIKEIILVDDFSDSLD
AYSLIRRHDKVSEQILGYPDSFLSEYIYRRND*
>BN1106_s6126B000060
MYERVVFLHPDLGIGGAERLIVDAAVAVNQSGYQVKMITNHYEPTHAFEETQKSEFNVIS
VADWFPRSVYGRFMALCAYVRLLLAAVYLIFTCNKKTDIIVVDQISAPLPLLNLAGFKTL

FYCHFPDLLLTDRSSHLKKMYRILIDYVEERSIGWASKIVVNSKFTAGIFRQTFSSLKNL
NLEVLYPVSSAANLHLPAAVFDSKNGQSPIANRQLCRKVLSSIDLPSSAKFVFVSINRYE
RKKNLQLILFAMNRLVQHWGEYFAGEQAGCATPQDVHVVIAGGYDTRVLENVVYYNELVE
LAQSLHDHSMRVIAATDCETAFTPVSYKTKFN*
>D915_12022
MRSDSFCLNQFTNIDTRNQCSECGVITNTTVLYLRTVLTQKDNLCPFVIVNETNMLFGDT
LIKLARTDADNLTLSSDHKCREVKQNHEDQAKVSNEEFEFSLAFFFAVYKEFNHFAGLCR
AVYRQRNAYCRHVDAKSDGRFRRQVEY*
>D915_04596
MKLFLSVLFLSDTFFSPGEHGLAYEVDRMFLLPVEQEEYDWGWKNNAFNQFASDRISVRR
HLPDYREGTCLTNLYPKDLPSTSVVICFFNECWSTLIRSVHSVLDRSPSHLIKEIILVDD
FSDL
>BN1106_s6008B000043
MDQKLSSKVKEWLEWDKNEATRSEIDRLYNAKDWKTLEKLLMTRMEFGTAGLRATMGPGY
SQMNDLTIIQTSQGLLKYAKAQFKNLNSLGIIVGYDMRHNSKKWAFYLANIFINAGCKVY
LFQDNYPTPLLAFGVRHCKTALGVMITASHNPKEDNGYKVYWSNGSQIISPHDKGISQCI
DECLIPEPASWKIDSVKTSPLCSDPMPELLQTYCELQRKALCFTP*
>D915_11873
MRILCFILTEPKNHQTKAKAVNATWARRCTTYFFVTSKAEPSLPNFVAVDHEGREMLWDK
VKNSLKHVALKYAENHDFFLKADDDTYVIMENLKKLLANMNSSEPFVVGRHFRLPRNRLD
YLSGGGGYVMSKEALLRVVHGIKTKPACGGSSRGGAEDVNVGLCVKSVGVKVLESLDEFG
LERFHPFDPRHMFNPETLRSIPWFHHFNYHKAVTVSPRMFI*
>BN1106_s866B000224
MELFNKSLLEDLAIDDLLNCSTQKFDGDSSKPVLRARPLNSNDYGYLDLLIQLTKVGSVG
RYDFEKTFSRMVACPETYFILVIEDETSKLIVAGATLFVEQKFIHECSKRGHIEDVVVDS
AYRGNGLGRFLIEALVRIGKHVGCYKITLDCHDDKVAFYKKIGFGLMNNMMYVRFDDHGK
S*
>D915_00062
MSDTQTSTIVDTDESIWEDENLNAKETKRIGPGEQGKSVQMTQEERKMEHYYYIPNGFNK
LVSDKIALDRSLPDIRHEKCKSMKYLRNLPTVSVIIPFFEEHWSVLLRTFISIVIRSPKQ
LLKEVILVDDGSTGRPELKGQLDDYLESNYPNGLVRVIRSATHEGLIRARLIGARQAIGD
VLVFLDSHCEVTRNWLPPLLDPIVRDYRTVTCPIIDAIDAGSFGYSARSGGPRGAFTWEM
YYVRLPKLPKDELFPEKPFDDLIMAGGLLAVSRKWFWEMGAYDPELEVWGGEQFEISFKV
WMCGGRLLDVPCSRVGHVFRIKGPFFNGTVPKGNYVARNYNRRPHYLEIDAGDLTEQYAV
RKRLNCKSFKWFVENCAPDVAQRYPLIEPPPAAFGDIRPVDNMTLCVDAADGSLIRLNEC
VRDSVNKSGLQNFVFTLHEEIHPVNRTDCIDSSRSGSRAIISLYPCFGGRGNQEFTLIPV
LNRPHRTVMIKHVPTTNCLESEIKTKSVFVNPCEMRNTGQQWIWESIDISRFNKTVKSPT
P*
>BN1106_s8167B000031
STGWLEPLLDRIKSNYTNVVTPVIEIINTEDFSMGITRSKDVQVGGFGWGLTFTWHMPPS
RDRDRPGAPYSPIRSPTMAGGLFSIHRDFFKLLGEYDAGMEVWGGENLELSFKV
>BN1106_s482B000295
MRLPSAYPPLVSRDVIVYLLFLHICASNQWWIKHPSESTSTDFDSAAQQSIYRLLDFLRV
SFPTHLRNDTLNRVREMFDFAYGSYTTYAFPFDELNPIDCTGRGYDHKNPDNINVNDALG
DYHLTLIDTLDTLAIMGKSDDFTRAVELLLRHLSFNRDNAFSYLRRPLGKCINSFQLSIR
VVGGLLSAHMLITDPAHTFGNLRPESYNDELLTHAHDLANRMLDAFEGSPTGLPYPRFYL
GSGRKDNTTTEACLAGAGSLLLEFGCLSAILGDPSYASIARRVVLNLWNRRSGVTGLLGS
TIDVNSGKWINRMSGLGAGQDSFYEYLHKTAVLFDDAQIGLMFNEAFATMRYHLRSSKNV
SSCLGADGMPLVYWNLDMYTGDRMNYWVDSLQSVWPGIFDHLQMFAVSVVVSRYFTLVST
GITSGTSSQDKYARAKCGFATIHSVQDKSQEDRMESFFLSETLKYLYLLFDENNPLNRNE
MDYMFSTQAHIFPIKRIRQLIKKFTTNPFQSNKNPPIPNSDRTLKTVSQSVRRSTSCSFL
SL*
>BN1106_s584B000346
MICCSDSGVTPVLILTVFVTCFGSSFVIGYNLGIMNLPGEVVKNFTSKYIKDAPSPAFMY

ALVSAVFVVAGAIGSFSSGILAESLGRRNTLLMNNAFSIAGAILTGPCVVAESPALLYVG
RIVTGFNAGISMGVASLYLTEVSPRDIRGAVGACHQLAVTIGITVAYILTLDQVLNTETL
WPLAVGLGAVPAAISMFVLPICPESPRFLFTNKRNEYEARKAFVKMNSKEDVDMFIGELR
EEMETAQQQPKFKFSQIFTRRDLRMPILLACLIQVQQQLSGINAVITYSSTMLRTAGLAN
DKIQYCVVGIGAFNVAVTVISVSLLERTGRRQLLLWPAIVLSLSLLVLTITVTLASQLPD
GNGAKTLGIVSAVFIFVYIGAFAIGLGPVPGLIVSELFRQEPRAAAYALSQGIQWLSNLL
VLISYPSIDNIKNFISRFVQGSPGADFMYALVSAIFVVAGAIGSFSSGVMAEKLGRRNTL
LMNNAFAIIGAVISGPCVFARSPALLYVGRVVTGFNAGITLGVASLYLTEISPRDIRGAV
GACHQLALTLGILIAYILTLDVILNTETLWPVAVGLSGVPAAISMFVLPFCPESPRFLFM
NKGNEHGARKAFVKLNSKEDVDTFIEELREEMEAAKRRSKFKCGQIFTAKDLRMPVLLAC
LIQIQQQLSGINAVIAYSSTMLTTAGLARNNIQYCVVGIGAFNVVMTIIALPLLERAGRR
QLLLWPAVVLALSLLVLTITVTIARQNANQPIGRTLGIVSTVFIFVYIGAFAVGLGPVPG
LIAAIGGYSFLIFLVFVVACWVVFFLYLPETKNRTFDDVAEELATPHIVVGKNHSHKEKS
SMQLFTRPNFADETQLGA*
>D915_15448
MAGPKNLGSRLPEQRTVPVKASKDTPSIGNILSEFWKSYKSNTPRRIQLLDAYLVYILLT
GIIQFVYCCIVGTFPFNAFLSGFISCVASFVLAVCLRMHTNPQNKHLFTPFCPESALGDF
IIAHVILHLVVFNFMG
>D915_14130
MRVILQRIYQFRNQYSYFYKADDDTFSIIENLKHELANHNPDDPFMTGFRWHINRHVT
>BN1106_s452B000152
MYLSFSAHRESKDLCRSDPEWNVVSLRYFNPIGAHASGDIGEHPNGVPSNLMPYVSQVAV
GQRPHVNVFGNDYKTKDGTGVRDYIHIVDLAQAHIVSIQLLDKKCGLKVYNIGTGTGYSV
LEIIKAMEKACGKPIPCKICPRREGDAAAVYADAGLAAKELKWKTKFGLDKMCEDQWRWQ
TKNPRGYE*
>BN1106_s741B000216
MTIRCTEVHHPFSFNRLPYRNFVGGVLAMSREQFAVIGGFSNYYFGWGGEDDDMFHRIKQ
HSFSVIRPKMTRYRMIRHGRDKLNERNKENLQVLTLTQQKALDNDGYLQSDYQILTAGPR
YSGLVYWIEVDVHESKLTAQRDALLKGK*
>BN1106_s554B000506
MTLGNFKLAERGSNVRFLHFVLVLQIQEAKCNFGDHLEIQLGDRPPADVNHPDLQNKLLL
LSLPSCSSRWVESPGLRGLMLAVMSAALISDLTSIFNSASTLFAVDIYKRFRAAATDKEL
TLVGRVFVVLLVIASIAWVPVIRELQGSQLFIYIQAVCACLAPPVAAVYLLAILSRRCTE
SGAFYGLLYGLFVGLIRLVLTIIFTDPVCGEEDRRPWIVGQFHYMYFALFSFLSTSIVML
VLSWLSTAPTPEQVHRLTYWTAWDTQADQQINLEVAGDNTVIRDHLVSLERVHGSENLET
LQNQPTNHASSVAGTTKYYTSDRSTDTTDAKQCAKHLCLWFCGCEERPCAPEQENRLIAC
CCCGRVRPFDETEESEGQLQGEEFRKCHAPRLQKIISLRQDPRALIGLRFGLLFTVLLSV
FGFAFFSLYFDPISPGPLPVIFEENSTVSEQVVLALGALQRQNLVQLIR*
>D915_14042
MHLRLRSVRRAVLVTSVLFFTSCVYLYLYAGYKENDPLESDRQFQLFQQKSETREKELLI
HNQFDPTEITAHRQSTASKMEMEKIFEELKFDNKPGGVWKQGFEITYNMSQWEREPLEVF
LVPHSHQDPGWIFTIDEYFEKKTRAGLDATLDILLRHPEARFIYAEMSFFSKWVSGLTPK
SKSLVAQLLHNGQLEIVSGGWVMPDEATASYYAIVDQVIEGHHWLWDNFAYRPNISWSID
PFGQSTSVAYLIRKMGFMGMVIGRVHYEVKKYLAQRKALEFHWRQSWDPETQAQIPCHLL
AFYAYDVPHTCGPDPAVCCQFDFLRLKVAPCPWKHNPSVIRAENVDER*
>BN1106_s546B000526
MFTYYLWVKAIKTGSVFWAAACALAYFYMVSAWGGYVFIINLIPLHVFVLLLMNRYSAKI
YVAYTTFFILGLLMSMQVPFVGFQPLKTSEHMASLGVFALIQAVAFIKYLQDRIPSSRLK
QLFAVSVVAFAGMVFLTVVGLTYTGVIAPWGGRFYSLWDTSYAKIHIPIISSVSEHQPTG
WSSILFDLHVLVAAFPAGVWLCFTDLNNERVFVVLYAIFASYFAGVMVRLILTLTPIVCV
FAAIAFSRMFEVFLDDEEEESGNPTRRSHETKNSSKTDKHLYDKPSKPIPQFMDTALIIE
STSQGLFVIGTHRRCICDTPPICKLKLRVSGSERVVKNVRLPPCRSEVGTPLIEAYYWLW
QNTKPDSRIMSWWDYGYQIAGMANRTTLVDNNTWNNSHIALVGKAMASNESEAYKTIQSL
DVDYVLVIFGGYIGYSGDDINKFLWMVRIGGGEHPNEIQERDFLTPQGEYRIDNAASSTM

LNCLMYKLSYYRFGEVRLDMRHPAGFDRTRNVEIGKKHFTLDYLEEAFTSEHWLVRIYKV
KPPKNVPSLKRPRRRIRTQQSTKPMNNMRGQLKFNSRIVRGKRSSTRERLFLSSINLAAL
EKQIGIQEPGFSRLARSSTETALFVSVPVLLIPAAVNGVAIVAVELPG*
>BN1106_s5191B000044
MSSELSINLSKTESLNLANSTRFTYYAAMGDYPIAPRMLGLVSFWDSHMVFNSRRILNML
SRVLRLFAVVLIMIMLFHLLRDQWLSPQNQSGKDSFLFRRTRLVSTEKDPFYPFTKFIWY
TFYRFIDPELLTVHTNATWYALSLFTDEYETMDPSARNEYFEDKLSEQLKRENITVQTGG
YYDPDGITDLTNCDKINQTLTILIPYRKRAHNLLAFLSYMHRYLPLKFVHYRIVVLEQIN
LKAFNRAKLFNAGLRELGVLINELKRPFTEDTSTLHNCTSYCFVLHDVDKLPVSMQTPYE
CSPYPFQLARIAVARNKSATWVGNHRLEKGKSEHWFYDSFFGGVTIVNKRHIHRVNGLSN
SFFGWGGEDDDFRKRMNKCSLSVQHASRNQSRYYFLDHPGDSAYNKRAAFLLRDHRVLIR
MHRDGIRQTRYTVRGRIVRPLYTLYQISV*
>BN1106_s4903B000048
MGFRLNEVSLLLSCSLGVFFSYLVYGILQEKLTKADYGPGEKFDYFFSLLLFQCIVNSLF
SAIAMFFNGEKKTTASEWDFAFCGFTYIGAMFTSNYSLKFVSYPVQVISKSVKPIPVLVL
HVLWAKRKYPIRKYIFVFMISMGVALFMYNGKTSITNQIGWGEALLMISLMLDGFTGGIQ
ESMRKVEVGSYTLMLHMNLWSIAYLSAALMATGEGVLFVGFARAHPEILPNMIYFGLSSA
IGQVSHSLAFTLRLIISIAEHCLGSVTLFPWVFVSPLEYVKISVYDLVCLPDYRQTASII
KAVFHRVTETKETLSVTGEQWYLTFIATHFEVHFHCKVSPSTQKLFVIFLFTLLTNFGSL
TCSIVTTTRKFFTVLASIILFSHVMTQRQWLGTMLVFCGIFLDQYYSKSKKVSVEKTENG
SLSKKQ*
>BN1106_s5966B000046
MWQRSKLPIHLSCSRVLLVFSVLLIGGCVLFLYLRFDINTDTTDHWSEYGLITNATLLYL
PTVLTRKDNLCPIVLENQTNMLFADTLVNLVRSDTGNFIPSDDQKCREFKQIHEDHVKVS
DEELEFPLAFSFNVHKEFNQFARLFRAVYRHHNAYCIHVDAKSDLIFRRKVEYLATCFGP
NVYVIPLKQSISIRWGDLGTLEAWIRCADFFLKQSVIRWKYMLNGSGQEFPLLTNWELVK
XQIHEDHVKVSDEELEFPLAFSFNVHKEFNQFARLFRAVYRHHNAYCIHVDAKSDLIFRR
KVEYLATCFGPNVYVIPLKQSISIRWGDLGTLEAWIRCADFFLKQSVIRWNYMLNGSGQE
FPLRTNWELVKALKAVNGSNIVESDYPNTGKSRVPKKPLSFNVTWFKSSIYTALRRDMVQ
FIFTNKYATGILALLRTEGHLSKLQDEVFFATLNYNPHFNAPGGCPEVHRPNQSDPRSAF
VTRYADWSPISCMSKRSQRGVCIMGVRNIPKLIRRPEFFVNKFIYDFEPLAFDCLEWWLF
RKIHDERDFGHTAIDFDPSFYHAKSDLIFRRKVEYLATCFGPNVHVIPLRQSISIRWGDL
GTLEAWIRCADFFLKQSVIRWKYMLNGSGQEFPLRTNWELVKALKAVKGSNIVKSFSTRE
TRRIPKITRTVIRIEPPCLLHLIQDNDCFSQ*
>D915_04455
LNGKAPHIYDRDPGAFPLDPGNRWATIDGDGDRLLYFYIPDAPDSTTGTGDEPKIVLLDG
DRISCLFATFIKRLLPQDRKLTIGVIQTAYANAASSIYLEHELGVPVVCVPTGVKHLHRA
AQKFDFGIYFEANGHGTVLYSSAALERARSLTPDHPLVVFVSLTNTTIGDAITDIMMVEY
ALAYLGWSLSDWAGLYKEFASRQLKVTVERPHLIQTVDAERRISCPAQLQVSPFCLVPLS
PPRF*
>D915_10349
MGLHFFILAPNTNQTPHLRKAFSPISESYTVKLRSPEEVLRYKHARWEALNNLDGRMFKA
NKNLGCESPRSSSNSYRQSARDSRGVFTGTKLFRFPRYVDMPKLLCRFKQGVSIFDDSIT
DRNFVLLVSPPLLCGYGAKKTIELLILIKSAHVHTNRRMAIRKLWGDDRCWGGRKVRHVF
LLGLLNNQTAPRLPQVDREIEIFGDIIQQGFIDHYYNNTYKMLFGIQWAVAFCPEAKWLM
FVDDDFFVNPRLVLSFIDSLDPRLQTKLVVGDLAVKAAVLREKSKWSVNKTLFSHRSYPN
FVQAGAFFMGAPMAVDLYVGSRFTEFFPFDDVFIGLVLNKLLVAPAHMRGLLMYQPNFRK
RLILNGSLALHGIRSAERQKWLWHIARLRDMCRVSK*
>BN1106_s513B000213
MLTKICGIGAGYVGGSTLIVLAKYCPEIQVTIVDISQEVIDQWNSDTLPIYEPGLDKIVK
EVRGKNLFFSTDMEKAIDEAELIFISVDTPTKKYGHGQGRAPNLTNLEAAARYVAKISRS
PKVVVEKSTVPIKAAETVTRILRMPASKQSTEEKNGINNDHSCNKLRESVVLSNPEFLAE
GTAVHNLTYPDRVLIGGDEQSVSGRLGIEMLRSIYLHWVPPERIMVMSTWSSELSKLAAN
AFLAQRISSINAISAICEKTGADVRDVSRAVGADTRIGPHFLQASLGFGGSCFRKDLLNL

VYISESLNLSEVASYWYSVLQLNEYQQTRFARRIVSKFNNTLQDKRIAVFGFAFKANTHD
TRDSQTIPLCNALLDEKACVAIYDPKVHPAQIVSDLLVANSVETVQKHVVVCSSAEEAVT
DAYAIVICTEWPEFKILDYKHLFSLMNRPAVIFDGRVLLNHGQLQKIGFEVESIGVDTTV
RKQRVVS*
>D915_15604
LQNTESFDPAQDDRCRRDALVKNKLFAWTPAHGECSVMCFHPRSDLTLALMTHDEVLRVI
QAWCDMTSEYRNAKSYRWLQIFENRGAAVGSSNMHPHCQVRSSSIRC*
>D915_06100
MFQLRFATLSEYFEAFYRRHGHKPRAATQSSVSPMLLNLTLFTGDLFTYADRDHDYWSGF
FTSRPVEKFLTRTLESELRSSELLYTYARHLIQRLPDSSLNETVHLLDDRITLARRALGL
FQHHDGVTGTAKSHVVADYNRRLRSALNDCRLISAVSSAALLLALPSPAADPTKRAVNPQ
QVINTIREVHRLPKESQGVATIISMEDLYFREAAPVPYRIKIETTHESMCVFLRVVFYCY
RCYFLLFPSVFVM*
>BN1106_s924B000329
MPVNPTLLNILFRFVVLTLFIIDQRQTMFCVFAFYACVSFCLAEELFSGPGLETDTCTAF
SQSSSKYLPVKKLHWSAKKRILVTGGAGFVGSHLVDRLMQDGHEVIALDNFATGARHNIA
HWLGHINFELLHHDVSDPIHIQGRFIWLEYVASCSFAFSFSLPTLDEIYHLASPASPPHY
MSNPIRTIKANTLGTLNMLGLARRTNAKFLFASTSEIYGDPEVHPQPETYWGHVNPIGPR
ACYDESKRLGETLTYAYANRLELSVRIARIFNTHGPRMQLADGRVVSNFIIQALQNKPIT
VYGTGKQTRSFQYVSDLVEGLVRLMASNYSQPVNLGNPQEYTVLDLAHLIKNLTGSTSPI
EHHPAPTDDPQRRRPLIQVAKEQLNWEPVIDLKEGLKKTLAYFRDYIDTLLP*
>D915_13029
LFRAVYRHHNAYCIHVDAKSDLRFRRKVEYLATCFGPNVHVIPLRQSISIRWGDLGTLEA
WIRCADFFLKQSVIRWKYMLNGSGQEFPLRTNWELDPGAWMKEKRNCAHEVDVIQVEVIN
NYVKSTYGGKIR*
>D915_02463
MTPMTDLDRRVNITNLLGGLTPIGVWSPIECMPKERLALILPYRDRDEHLRVFLNHMHPF
LRHQQLMYTIIVVEQVSLQLPSVTEHVCFSSNDPFCVDFKDTRFNL*
>D915_12525
MNNNPRILGQLCLGVLVFQNLFYTICMRHARSRGEEQFFPSSVMLISEALKMLTCILVVH
FTDGLWPSISYLRNNFKDSLKTCVPALVYLIQNRLLVAALECLDAATFQVAYQLKLLTTA
LFSMLILRREISFMQWVALCLLFIGVSVVEPPTQSVPSETLNPSLGLIYVICAAVLSGFA
SIYFELVSPHVCFFRPHVVNPKASQKFVQIAVVAKFGTC*
>D915_15410
LAFLSFAFWRWQVWACGFLPSLVERYDRNQWEYFADHNIPLLLDYARQEEVGMSQSDCRI
VTYNEHWLVVVPWWACWPFETLILPRKRHIRWLDELAADEKGSLTRVIQELLIRYDNLFH
TDFPYSMGWYQAPLHHIGCDVLTPKESYQEQAHWQIHALFQPPLLRSASVRKFMSGFELL
AEAQRDLLPERAAEILRQVPTVHYTLTATQ*
>D915_14055
MTDESLFSSKEEAKEFLRRLCDQEIVVHVTDGRRYVGRFCCTDRSANLVLGSCIEYPAPA
DATYEQLQRNLTAVVIPGQHITKIECDRNLLLLPLTSLKSWCDITEMDRRKAELLYIFYV
VSAYWIVSISLVFVNKWLLSSRTVSFDAPLFITWFQCATTAILCHVTSHLAYIVPSRVQF
PQLDFTLSTAIDASYRPFTLIGAFIYPGVTSFCCVCLHGHVQ*
>D915_01605
MCSTFKITPYVPFEYTVTGYLERLNAVISRQSFCTGPERLHLLRTPWELDVSGSVRRQYL
ELDANVIHWPPIGSGGVRFILGPPDASCVDSCATATLPTGDIQLNSSYLHRRAADYSVRP
FHKLNASMFCAPEQFTSANHYALIESLLPPGGRCALIQSEADSAAPSLDPTGEVCILQAD
RSLFDCTAKPTRPGVRRLCPCQIGLPGQTSLCIGCV*
>BN1106_s6854B000028
MSRRRGNVETTVFATVGTTSFDDLITEVNKPLFHAALWCIGYRQLTGDHGSARQSTHTNP
REMSRRRGNVETTVFATVGTTSFDDLITEVNKPLFHAALWCIGYRQLVIQFGDGVVEPKS
PSFESVRDAARYSKMCVDANVKPLSIDSFRFKSDLDLEFSSSSLVISHGGAGTCLRALTP
GGLRRLIVVINETLMGNHQEELAEALAEGRHAIVTTPSKLLKLLCDEPGGCKFPGKLSSS
KISELLRPQVRPADAGFTSFQRGSPERLLEYLETRLKT*

>D915_08355
MRDFQLASSFRSVFLIDEAYFSLTKVSYPFRAQRSLHRITFTLPTNGFPNRYWGWGNEDD
ELAARCYLHGLQLTRPRTFVGRYRAVRHLKATRGSGHYDSFRAFRNFLRDGLSALSSTTY
RILEDSSPERLLHHTPQTKTVANVCNYSKLLTAFNITMSDWLEKACAPKGWADRWDALAR
LLLYTHLVVDVDLMRYQTIQPTSKSRESWYWFLHFYGWI*
>BN1106_s933B000129
MIKIRLKLFRMKFVQTFLSALAVLLLLLCFYQAKKLFKWGQFRLLLPYRTAPIDCSLSYG
NFIISHSSAHQPEEIEFPLAFSLVVHTDSDRVLRLFRAIYRPHNYYCIHIDRKSAPTFVE
EIERLQQCTQLSHNVYFVELSDRIDVRWGRISVLDADLACARILLNRAPNRWKYWINLTG
QEFPLRTNWELVRALRLLNGSNLVESIYKRRNIDRFPPRKRTPFNFTWHKGSVHIAVRHE
FVHYMFNNPKGEQLYQMLREHEINTGKGTVPDETYYATLNHNPTVFPIPGAFTGVHEQHV
TIPLARAKIWIDSNNQICGSGHWQRTICVFGLADLPFLFSQPHFFANKFLPNVEPLAYDI
LEWWYFSKVRNESMYGRLADSFNSTYYLLSPYAKQHLM*
>D915_08493
MQRFQPLSEEKNAVAVSTLGRQLPSSDRHKSERLVVSPIVYDTSVYGDINRSLYLGGFTW
NLTFRWEYPPTDTLITKSHFDPQSTPAISGGIYATWRDSFFQLGGYDEQMQIWGAENIEL
SLRTWMCHGRLEIIPCSRVGHLFRDKHPYSFPDGIEHTVVRNRKRVALVWFTHSDERVTS
GIRTNLQSYLSRFYAASPTALQVESGPIGDRIDLAKQLKCHSFDWYLNTVYPKLLDESEL
NVEF*
>D915_00809
MGAEISAPRPLTKFPSLNTLPLSLERKRAMSKGRCTCGNPLYMDSSEFGSGRRSSVKIQP
ADEKLFQKVMEIMKPFELSVNQYNHFCDLMSETMDKGLKLATHQEASIKMYPTYVSKIPD
GTVESGAYLALDLGGTNYRVLLVHFSGKSTLPRIEERTYAVPHSKMSGTGEELFDYIAKT
LADFLKSHGVAEKRCDLGFTFSFPCEQKGLTDAVLVRWTKGFSAAGVTGQNVAQLLQDAI
DRAGANAQCVAVVNDTVGTLASCALEDPRCAVGLIVGTGTNAAYVERSENVQLMDDKSNE
FVVINTEWGAFGEAGEFNALRTRFDKSVDTESINPGKQL*
>BN1106_s626B000340
MYGMYGMYSCKQEGSDCLINSYRHILEIVLLVTMSAIGIRLLKKMIFSYRGEFLRAGFAG
TDMSKSSRPVLPEAQGVLAGAVYIAIMFLFIPVPFWRHLFGRTYFLPVVEVRQDVAFILD
VIGQCFHHHYIPVGSAVQKPSVLYYVYMGLLAVFCTNAINIYAGINGLEVGQSIIIACSV
ILFNIIELQGQLTPYPPRRF*
>D915_05925
MLPEDTEHVRETVRRLLSTPVDSGYIPFELTTTGYLERLNALLNHQDFCTGQASNQSTTF
TPILAPAGWSCNKACESVERLSPPLTVLSGPQTTKESIQVVPSSYLDHRYEYARPRTASI
KWQRLRCAPQYFTELNRADKLSQLWNFTCRTNVPSADMTAPFVDVTRGACSFQLNNLRFS
CTYPDPSDPVKPNIQRICPCHTRLLGQSTLCEQCV*
>D915_11750
MKTLHSRRHQVTCVEPTSSTFSALFRPSFLRVSPSNHRCHFVDDLHTKLESYVSKLPIHV
RIERMPIRSGLVRARLRGAANATGKTLTFLDAHCETTVGWLEPLLAEIAVDRRRVVCPII
DVLDFETFHYSEGSDRIYGTFDWQLTFHWSPISSSESKRVGTNHSIPIRTPTMAGGLFTI
ETDYFHEVR*
>D915_14141
MTRKHLTVMEWVSKGYCESLEYLIKVDDDTFVDVFHLVRFLKTERLKTPNSFYCSATSGA
RPIRPSKKTPSKWVITTTEFEKSVFPTYCEGLGYIIEAHLAPYLYWCSLFTPPIWIDDVY
VTGILAENLGFQLQEFIPGHAYSRVGPSKQNEHLLDSIFLTSYHSEFLPETFRRLWQTAV
SRSMDIF*
>BN1106_s5930B000044
MLFRNILTLGKLLICLNVSLFLCPIQIVQSRPLKSPTFDQGQICLNCECWMLHQLNGLVH
PHPRTISDLQSWLPKVSIVIVFHNEEPANLLATVFSIWNTTSTNLIKEIILVDDFSDSLD
AYSLIRRHDKQKFIRNPERFGLIKSRMVGARAATADVLVFLDSHVTCTTYWLEPLLVRLV
SSRMRTNLMGASGEQKKADDAGELQFMCEFDANEENNIDVRFGCIGTDFSEMLCSA*
>D915_02926
MIITAAAYFKTVPSHSDVHTPDYVTRILRRSFLLSTGADCCSDYSVSFHYLKPLDMITYD
YLLYQLRPYGIHHDYNDVVKLLRNHSAFI*

>D915_12914
MGVMIFAVFLVITLGTFFQFGYHNGVINQPLELITQFIENVTIERHGEINGTPLTLLTSL
CVSTYLIGGLFGSLLGGILSNKLGRRTSIIILSVPCVVGSVLLMLCKWAHSFEMIIIGRL
IVGLACGAFTAVGPAYLYEVSPHTVRGAAGSLNQLVLTTVFHINTVSVGLNAVPCVLTAV
CLLFIPESPRFLYIVRNDVESAKAGNICTPTLTFFQTNSAFFKIGRSPEQVEFELDEMLR
ELETSQHKISLITFFRTPHLRWGLLVALVCQVGQQFSGLNGLLYYSGTLFTQNGLTTEQA
TYATIGLGLALFFSSLVSTLVMDRLGRRVLMIGGLLISFFSLIVFTVCLIIHDSIGAQWP
VYIAVAATYVFVIGFGIGPG*
>D915_04802
MRRHRFTKKLNQFRYPFVLVVDVDVSAYQSIEIARTAAIRVTNMCALGRSTRIFSFRGFQ
CSLFHMAATEWTCDIDVSSPVLTPPSRLSTERMVFSTSTDHNALPVNTSDIEPATKRSRL
TLTDEFTRANGVSESYQKPVSDSAAARLGLSDRFKISFCARFKSALREKLESYFSRLEKA
EESANTIQKELTELSGRMDAIDTSSQELKKFVTERLLLLVSNRMSRVLSLCSPTSIDLNN
AAADTEDREHLWTKTKNGLHHAVSHYSDYDFFLKADDDTYIIVENLRFLLKDRDSEIPII
MGRRWRNFRGDGFLISVSTRLTVSLAGTRLHLPQECKDYSRGINGRRSSDSSKTGLNPLT
FVARCGQPHDKAIMFEPTDKLLVHNQAQGYLSGGGGYVMSRAALKLIIAGLKMDPSCAGT
EAGGAEDVRLGTCAEKVGVKLVDSLDSDGYERFHPFSALGMVNHVNSDNPGWYKSYNYHK
ILTLADNLSLTYYVRKTSKNHNFPSICACVCVQIEMEMSRGLLYGTVRQLIPLGALT*
>D915_04456
MHGFCLCLNLVTTPQLHYAVYHMNQRTQHESPRKQPVVPDLCQIYVNRFTTRFCRGLDGL
KQMKESSPVQPVLLNIDCANGIGSKVLSLVRHEMTNSDCPVRLQLYNTQTKRSDWLNKNC
GADFIKVCWLSINLLRNESYLASVERVFSQALTSFIWGF*
>D915_05377
MRLLSKEEASPWFMRILNCQIFSYIADTLRDFLQERGMEAEYLKMAFVFNFPTEMHGINE
GVVVSFTKEFECPSLIGQEVVGGLQDAIRKLGLRIEICALLNDTVGALAAGASRDPDCYL
GMILSSGVNCAYFEKLTNIQYPVNLGKEVDRVALNTEWGALGEDGCLDEYLTEYDREVDR
QSFCPGKQM*
>BN1106_s855B000399
MGGGARGDKKTKRWNPNASKPGEGNTHSYSSTAIGILAVSMASVSSGFAGVYFEKILKGT
APSIWIRNIQLAIFGIAVGLFGVYTYDGKAVIEKGFFQGYTLLAWLVVALQTCSGLGVAF
VIKYADNILKGFAAGLSIILSSFVSYFLLNDFSPSTFEYQMLLSRLFASEVDYDATDTYV
ISTDATGALFGIAESHRS*
>BN1106_s5430B000048
MARSLLRKRKTIVIFMLTIPSIFLIWTFIAQSQVPAPPAAPSRKSFDRQNFRKSKFQLHV
DQKIPEFPKVDEIHEEQKAAEPPKAPDVPRPSESFVSVQIFPSAEKPDKLIKPDNGGPLQ
MKPPVLPSKLNQSSGESGKPFLIKKDSLSPDERREYDQGWEHHAFSQYASDRISVRRYMP
DIRDEGIGESGKPFLIKKDSLSPDERREYDQGWEHHAFSQYASDRISVRRYMPDIRDEGL
VDPLGKPPLLADAILGETMEFGTDVNFTIFGLVHSE*
>D915_01291
MAERNLPWLPPAVPQDIAPELHHYHGAPFVWFMGQLITYLMRPSRAFGETMNKLFDQYQL
TGPKRLPTVGIHVRRTDKINTEAAFHDLKEYMNYVDRYYEYLEAEQQMMARKEEWVDDVF
SEHRLRTNPIKRRVFLATDEPSLFEEAARAYPKYEFVGDASRAETAAVVKRHEANSVTGI
ALDILALSRTDYLVCTFSSQVRYRLMHFSPDLCA*
>D915_07894
MIWQTSSNFCIMSIHLQNAMLYVFGVLINGLAFATDVLCSDDNSASWNVFKGFSHWTWLL
IITQSVSGIFMGFVMKFSNNITRLFIISSAMLVTTFSAMLVFSLHLNAYFVASFILVFTS
LYLYHF*
>BN1106_s7879B000034
MFFEEIQFHFLMRINFYSIRTWFMFHLASCHELKQVSILAFLFLQILLSNFLAQTEALMT
GKTREEVHAELFSAGVTGEKLEALSLHKSFKGNRPTNSIVFTQLSPYMLGALVAMYEHKI
FVQGVIWDINSYDQWGVELGKVLAKKIQPELKTPGPVTTHDSSTNGLINFLKESRK*
>BN1106_s547B000277
MSCRAITLRNLKQISVIICGICVLLLISSTVQPYLKEIYNKNISNQYRLPFSLKAPLQQL
DDGLSLGLNEPVAKPFCSEKVKDLVGRIVINLEKPTWDELIVRFAPNGTIDQMNIMTPMT

NLDRRVNITNLLGGLTPIGVWSPIECMPKERLALILPYRDRDEHLRVFLNHMHPFLRHQQ
LMYTIIVVEQSCSLFHTSYCRLMYPELFGGAVALSRDHFKKIRGFSNVFFGWGGEDDDLY
YRVKHHHYRIFRHPSHIARYTMIKHKRDRSNPANPERSACTMHRLLKSSSQRFSTDGYPE
SQYTVKFAGPKYDGLVYWISVDLDENQVLRRFVRVHRLVTVFSVFLRFLRSFLSVFLKRV
VGVFTARIFLARLVSQFSFQCVV*
>D915_14023
MKRSLQNKWMFYAEAIVSLMFITGASLFLAFVFSEVQTEPYMDEIFHVRQTKSYLSGNWT
DWDSKITTPPGTYVLFLSVYRLLEKVHFIPDASLISYMRYFNAFILSLNYVLLLGIIKCF
DGIQSPILTGLSIITNPVLFFFSALYYTDQCSLLFILLTLYSSLCGHGILSAVFGSYGIF
VRQTNVVWLLISLCILVGRLIFPLYASNQDASILPAGIWFRRILLKTFRQPIVIWQIVCH
AVSRAPAHFVAVLGFMFFVHHNGGIVLGDRSAHRAVIHVCQLWYFVVFCTLHTPFEALQF
LRTCFRRWYRSSYTDLVRICLTTSLVLALVISSLRYTSFVHPYLLADNRHYTFYIWRKVI
SRSTLTFYSLSIVYLFCSAYWMDCLFGSGLTFTHWLINMGLILGSVLSLVPAGLLEPRYF
LPPYVLWRLFAVRFRTVSCRSWFFEFGLNAFVVVITSYLFCAKPFFWTHEPGVQRFIW*
>D915_14859
MPTSTLGDGVLTDGTKKIKAIILIGGPCKGTRFRPLSLELPKPLFPIGGFPVIYHHIEAF
SKLPGLCEIVLLGFYQPHESLNQLITNAQREFKVTVR*
>D915_11092
LRANILRLSADYVRLLAREQQDTADGPYRSLGTNYDMKKTMAVLLNEMNIRVEQLDAQVK
ELSARVTGSGTESRSLSPSQSQRRLHQIGGWLRARWRSDPCYAELGVDGTDCSLVRYLSE
VENFCPFTEDKLKYSSRPFAQYLCATVGLSPEVMLTE*
>D915_15745
MNLAYTNAGSQKHLRPIFFSLHTVLLSLSYLLRGQDKQFIVLSGIAILVFRSELILFYGP
CLLYGLIQGSVRLRPALLLTVFMTAVSSVSLFLSPFLGPDIFFSSRECNFTRRGDESGDN
GLLSVVKSPFHWYFTSALPRALLATTAMLLCWFPFALRSLVLSWRGRPQIHFQPMSTGLI
FVGLVFVSLYSVLPHKELRFIIYTVPVFNLAASVLWNFLLCRIRVVDFPNLVRHFSEHSE
SRLRAVIRQKGNKNGRALSKKYSAFRFCNWLCYAHLLMNLIGTAILLVAARKNYPGGHAM
MRLNEVPSLIREPHIHICNLAAQTGVTRFIEEHENWTYNKTEGIENDVHLLDHSLFTHLI
SEIPTSVLQQQSDKFRPLFFVDGFDGINLQLNWTTVWQFIQFRTSPRLIVYERIK*
>D915_02927
MNLSCIAAGVNESRDSLWDKVKFGVQMVMDNKPNDFDYFLKADDDTYMIMENVRMMLDGL
NPEQPFIVGRRFKVGSGDLVFAVYLIASIYDSNEKIQMHL*
>D915_03610
XFSMEFMVPVSYQSPIGLRGNICFRVAFLGAAVIGARVWTTTPRLKTHVFSMQLGCLFTF
TGYMFLWTSSRNLSTYWSSFSLFIVGLLFIGLAYPFLFCSAAIHLYESAPEAWRGTFGCL
PWLMITVAATLVQITITRFPWGLWISVCSSLAFIANFFLSQISEPDRQLMQKTDLSDAQP
LIESNDTENSTKSDSNANPSEVRFLIADQSDFGCFSQTRFQTLIWLMVMHHITGASAVMY
LAELMLEFAPKLWPVDYAVAIGLPQVIGVFLACLFANRVPSVYLLRFSSVVMTGTSFALG
YLLKQPQMKPINSCLITCIAFGLLAYAIGWGPIPWLLVNQMHSTADRRWAMGTAFFVSSI
ALLIVHVTFELLITVLGIGNYFWIVALICLCSTFHFRMPRFTNRGSVKILPRPNVKLCLH
PVGQALRVDPESRVHLLNRKRFLLGPRLKVV
>BN1106_s8380B000026
MPLSRHQQKVLDHLVQIEEVGELVLAKREQCVSCDRSRQKTREAIRALTXSSALGSDRPP
CFTSSHRRYNPLSGDWVIVPPRRIDRPWSGHTELPKAGKTAVNNEGEGSAKNPLSPGASR
SSGIMNPLYTGIFKFANDFPALVMFFRLLDNIFLSLFAPLTMSVGRMPPKRLKCTFTLKN
DQERFTIAFRIIFCYFFSRDNIHRKNPDGFVKVGLPLDDAIITLCHTGYFVSQYQNISVN
NRLTSLILYFVQFRALYS*
>BN1106_s7025B000054
MLLSHYLRILRSLVFQSRSIFLLIGFMVGCQLTFLSFFSLSRLVWMQKSVSLDNGSVEIF
DPFGRYGETVIADRLTNRIRIVCMILTMPSNHRNRSLAVKYTWAKRCSSYFFVSTKDDPS
LPAFGGFFPTRYQPPSGTEVMIL*
>BN1106_s773B000385
MVCQVNASSMSPKRRRFLWIVFITVALTWIILITTYLRQFRPTVQSFSWTICESLFQGKY
DLALQVQNELVPNRRLNSCEVIATFSGHVPWISPEEYRYPLAFAISVFENPEQFAHFLRL

IYRPQNVYCIHIDRKTERSTTEQFENIAHCFGPNVFLIPVDQRVDVAWGYFSVLQSTLLC
AEHLLNQTVVPNWKYMLNMNNKEIPLRTNWEMVSALKALNGSNLVESVPCPDLEQRKPKH
KYTFKFDWHKGSFLTALRREFVKFMFTNPNALELIESMRRESEQRKVQDELLFSTLAFNP
HLGAPGACLHAHRHNSSDPRSWFLARFVNWNASNCPSGYAVHGVCIFGAEDVPILIRQPH
LMANKFFWGFQPVAYACMEYWLVKRLRYEKAYRTLHPSFDRRLYANLYCSSDHI*
>BN1106_s584B000348
MALFGGQKLTFTLLLTVFMTCFGSSFLIGYNLGILNLPAPFIKAFLSKTILKKDIVEAEK
EKKFVNPSFLYAQVSTTFVVAGALGAFSCGWIAELLGRRNGLILNHVFAILGGILCGPCV
IAEQAWLLFVGRFVLGINCGITIGIASIYLTEVAPRELRGAIGACNQLAVTIGIVVAYIA
TLSYTLNTQTLWPVSVALGAVPAFISLLVLPFCPESPRFLFMKKNNEAEARKAFARLNVQ
ENVETFLGELREEMEVAKNQPEFKFTQLFTQRDLRMPVLIACLIQVLQQLSGINAVITYS
STMLRTAGIPDEYNQFCVTAIGVLNVIVTIVSLPLLERAGRRTLLLWPTVALAISLLLLT
ITVNLATTLSDAKAAQALGIVSALLILVYICGFALGLGPVPALIVSEIFRQGPRAAAYSL
SQSLQWLSNLLVLCSYPSINEAIGGYSFLPFLVVVVVCWTFFFLFMPETRQRTFDEVARD
LAFGNIVVGKRTATLEDRNMTVFTKQGERNVSNVDPATVQPLLQPSATRIDPTSYS*
>D915_11977
MIVPLLYACSLAFLLGILFVIVVVPWTRYIRRPRLREALRHQYGLSADFSLIAFFHPYCT
SRGGGERVLWAAIKSMELIKGKPAILVIYTSDSNCIKNRAQVLREVRQTFGVQLDAKASI
HFVLLRGTLLLSPQLYPVLTLAGQALGSVLVCLEALIRCPPDIFIDTTGFAFILPLAKRL
FGAKTAAYVHYPTVSSDMIDRVSAGLHRSENDLAGETYNNASWIRNSAFMTRIKLLYYRS
LIGAYRWVGSPSNTDCVMTNSTWTRNHILSLWNGDPSIVYPPCPTEDLASGDSDERQPWI
MSVGQFRPEKNHELQIEAFLQFLTRNALGTRADGHPYRLLLIGGCRDATDFARVDQLRQL
VRTRDLENVVQFHINVSYSHLKRYFHQCMINLHTMVDEHFGIGIVEGMAAGLITIAHNSG
GPKSDIIGPARASCSDQDQNVSIEPVSVGFLARTVEEYALTFEHVMMRMSDAQKDAMRKH
ARLWVQKKFSELCFSKSWLNNMQDLGL*
>BN1106_s938B000243
MGSSSAASIRLSRELTGDVFRRRLALVSLTIQNTAVIMVTRYSRARGGDMYFASTAVVMS
EVVKLITCFLLVFMEENFSFSAFRTNLKTNIVQDPWDCLLVSVPGVVYTVQNNLLFVGYS
YLPAVSFQISYQLKIFTAAIFFRIILKRELSRTQWFALVLLFLGVSLTQVSDVTESSAGQ
QEVATTFFQQMLALASVIAACMCSGFAGVYFEKLLKGSHKSVAVRNIQLSFYGVTAGLLT
VLIKDGSGVQQRGFFFGYDHVVWASVLIQSLGGLLIAATIRYADNIMKGFAPSVAIVLTF
ILSILFFHFTPSVMFVCGGLLVVVATVLYSICPPPKLVAGATRNASQSQSSSVSVKEAPA
RA*
>D915_15783
MRITLRAIYEERGSIMYFLKAGQSDYIIMENLRHMLLSEDPEKPFLIGHVKDPHDSLLSV
SGSAGYVMSRGALDLIVTLGLDNHPACAATDAEEDAQISLCAQSVGVQVRDSFDFLGKSR
FSNVSIFDMLGPFPNNTPRWYPQETDYTLPTFNLSKLPASPLLVSFTGVEGTRMYVLEYL
LYHLRAFGIKHRWSRDNCGKSLDRT*
